# Reactivity of Diaryl Bismuth Cations toward a Platinum(0) Complex: Oxidative Aryl Transfer

**DOI:** 10.1021/acs.organomet.5c00441

**Published:** 2026-01-06

**Authors:** Johannes Schwarzmann, Cissie Slopianka, Crispin Lichtenberg

**Affiliations:** Department of Chemistry, 9377 Philipps University Marburg, Hans-Meerwein-Straße 4, 35043 Marburg, Germany

## Abstract

Reactions of three aryl-substituted bismuth cations, [BiPh_2_(SbF_6_)], [BiMes_2_(SbF_6_)], and [BiDipp_2_(SbF_6_)], with the Pt^0^ complex Pt­(PCy_3_)_2_ have been investigated (Mes = 2,4,6-trimethyl-phenyl; Dipp = 2,6-di-*iso*-propyl-phenyl; Cy = cyclohexyl). Unexpectedly, and in contrast with the reactivity of the recently reported methyl analogue [BiMe_2_(SbF_6_)], the formation of isolable metal-only Lewis pairs [(Cy_3_P)_2_Pt→BiAr_2_(SbF_6_)] is not observed (Ar = aryl). Instead, an unprecedented bismuth-to-platinum oxidative aryl transfer is witnessed to give the Pt^II^ complexes [PtAr­(PCy_3_)_2_(SbF_6_)], along with the suggested bismuthinidene intermediates BiAr. Attempts to trap these fleeting intermediates with an *ortho*-quinone led to a Pt^II^ semiquinone radical complex.

## Graphical abstract

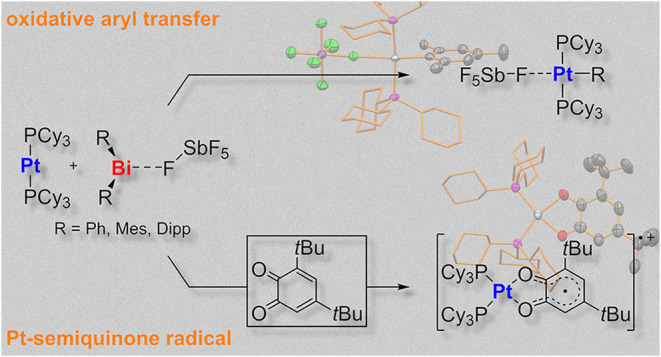

## Introduction

Metal–metal interactions in dinuclear organometallic compounds show a multifaceted and broad spectrum of characteristics in terms of the nature of bonding, bond order, and bond strength, resulting in a diverse coordination chemistry and well-tunable reactivity patterns.^1–12^ In bismuth chemistry, compounds containing Bi–TM interactions (TM = transition metal)^13–18^ have created cases of trapped reactive species,^19–21^ metallacycle formation,^22,23^ selective CH activation events,^24^ electrocatalytic CO_2_ reduction,^25^ reversible elementary reactions,^26,27^ as well as catalytic applications in cyclopropanation,^28–30^ O_2_ activation,^31^ and radical cyclo-isomerization.^32,33^ The analysis of Bi–TM bonding interactions reveals three major scenarios that may be distinguished: covalent Bi–TM bonding,^17,18,27^ Bi→TM dative bonding (with the bismuth component acting as the donor, covering Bi^I^ and Bi^III^ species),^14,34,35^ and TM→Bi dative bonding (with the bismuth component representing the acceptor)^36–39^ (Scheme 1a). In addition to the bonding schemes of Bi–TM complexes, fundamental reactivity patterns should also be discussed (Scheme 1b). The oxidative addition of Bi–Bi, Bi–C, or Bi–X bonds to electron-rich transition metal centers typically leads to the formation of covalent Bi–TM bonding (X = halide; Scheme 1b.I).^26,27,40–42^ In contrast, adduct formation between suitable bismuth compounds and transition metal complexes has been reported to result in dative bonding scenarios (Scheme 1b.II,III).^14,36–39,43–45^ In the context of fundamental reactivity patterns, redox-neutral transmetalation events (i.e., ligand exchange reactions) also need to be mentioned, which do not necessarily invoke any Bi–TM bonding, but play a key role in utilizing bismuth compounds as aryl sources in transition-metal-catalyzed crosscoupling reactions (Scheme 1b.IV).^46–49^ In our efforts to investigate the reactivity of cationic diaryl bismuth compounds toward an electron-rich transition metal precursor, we have uncovered the oxidative arylation of a transition metal complex as a new facet in the reactivity of bismuth compounds toward well-defined transition metal compounds (Scheme 1b.V).

**Scheme 1:**
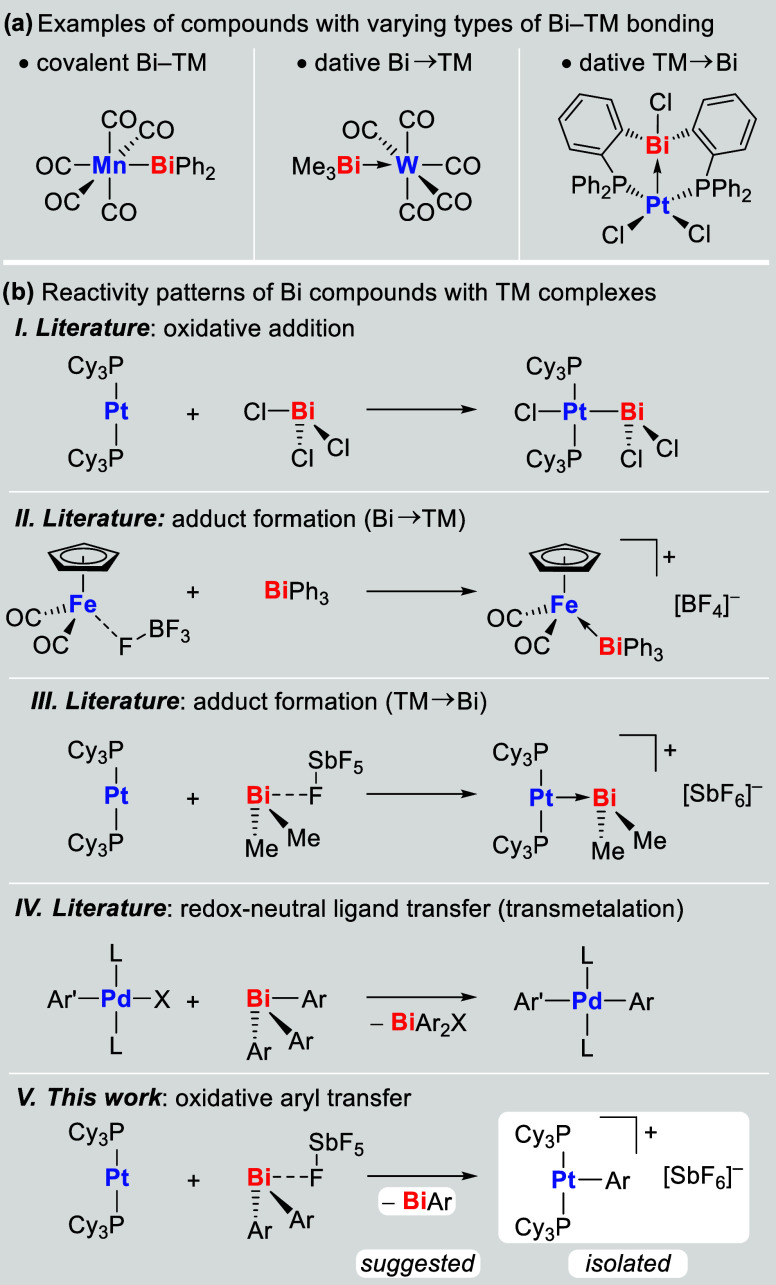
(a) Bonding Scenarios of Compounds with Bi–TM Interactions. (b) Reactivity Patterns Involving Bismuth Compounds and Transition Metal Complexes. Ar, Ar′ = Aryl; X = Halide, Acetate; L = Neutral Ligand or Vacant Coordination Site

## Results and Discussion

In the quest to further elucidate the parameters that influence bonding interactions between transition metal Lewis bases and bismuth Lewis acids,^18,36–38,50^ the electron-rich platinum complex Pt­(PCy_3_)_2_ was chosen as a model substrate to ensure a reliable comparison with previous results.^39^ In view of the apparent robustness of neutral aryl bismuth compounds such as BiPh_3_ that can conveniently be handled under air compared to their alkyl analogs such as BiMe_3_ and BiEt_3_, which are highly air-sensitive or even pyrophoric,^46,51,52^ we aimed at cationic complexes [Bi­(aryl)_2_]^+^ as potential bonding partners. However, compounds of the type [Bi­(aryl)_2_]^+^ with simple hydrocarbons as aryl groups and sufficiently weakly coordinating counteranions are surprisingly rare. In fact, only two compounds of this type are known to the literature, the catalytically active parent compound [BiPh_2_(SbF_6_)] and the strongly Lewis acidic [BiDipp_2_(SbF_6_)] (Dipp = 2,6-di-*iso*-propyl-phenyl).^53,54^ Even though other diarylbsimuth compounds like BiPh_2_OTf are accessible, they are of minor relevance in this context due to the (OTf)^−^ anion being a significantly stronger donating counteranion than (SbF_6_)^−^.^55,56^

In order to expand this series of reactive compounds, the synthesis of the [BiMes_2_]^+^ complex cation was targeted (Mes = 2,4,6-trimethyl-phenyl). Attempts to isolate [Bi­(Mes)_2_(SbF_6_)] from salt elimination reactions of BiMes_2_Cl with AgSbF_6_ in dichloromethane led to the precipitation of a mixture of [Bi­(Mes)_2_(SbF_6_)] and AgCl (Scheme 2a, left). The formation of [Bi­(Mes)_2_(SbF_6_)] could be demonstrated by single-crystal X-ray analysis (proof of connectivity only, *vide infra* and Supporting Information), but the separation of AgCl and solvent-free [Bi­(Mes)_2_(SbF_6_)] in weakly coordinating solvents could not be obtained. Using the same synthetic approach with a more Lewis basic solvent such as THF allowed for the isolation of analytically pure [Bi­(Mes)_2_(thf)_2_(SbF_6_)] (**2**) (Scheme 2a, right). After removal of the precipitated silver salt by filtration, **2** was crystallized from the THF solution at −30 °C. NMR spectroscopic analyses show the expected signal patterns. The relative integral values and chemical shifts of the signals due to the THF units indicate the coordination of two equivalents of these ligands to the central atom, with changes in chemical shifts of the α-CH_2_^THF^ groups by 0.02 ppm (^1^H) and 2.4 ppm (^13^C) compared to free THF.^57^ The ^19^F NMR spectrum shows one broad resonance without resolved ^1^ *J*_FSb_ coupling, suggesting bonding interactions between the Lewis acidic bismuth center and the (SbF_6_)^−^ counteranion.

**Scheme 2:**
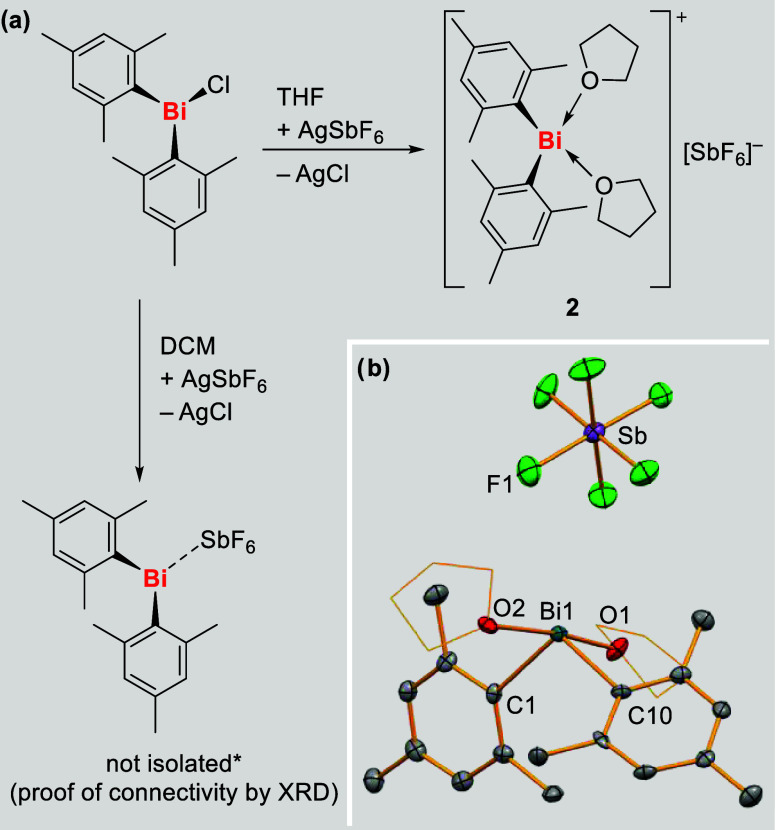
(a) Synthesis of [BiMes_2_(thf)_2_]­[SbF_6_] (**2**) and Attempted Isolation of [BiMes_2_(SbF_6_)]; *: The Solubility of [BiMes_2_(SbF_6_)] in Weakly Coordinating Solvent Was Low, Prohibiting Separation from the By-Product AgCl in the Absence of More Polar Solvents Such as THF. (b) Molecular Structure of **2**. Displacement Ellipsoids are Drawn at 50% Probability Level. Only One Out of Two Chemically Identical, but Crystallographically Distinct Molecules in the Unit Cell is Shown. For Clarity, Hydrogen Atoms are Omitted and Carbon Atoms of THF Are Shown as Wireframe. Selected Bond Lengths [Å] and Angles [deg]: Bi–C1 2.273(8), Bi–C10 2.273(7). Bi–O1 2.469(5), Bi–O2 2.454(5), Bi···F1 3.692(5), C1–Bi–C10 108.9(3), C1–Bi–O1 81.4(2), O1–Bi–O2 171.21(17)

In order to elucidate the molecular structure of the diaryl bismuth cation in the solid state, compounds [BiMes_2_(SbF_6_)] (monoclinic space group *P*2/*c*, *Z* = 4; only proof of connectivity) and [Bi­(Mes)_2_(thf)_2_(SbF_6_)] (**2**) (orthorhombic space group *Pca*2_1_, *Z* = 8), were analyzed by single-crystal X-ray diffraction. The donor-free compound exhibits a bisphenoidal coordination geometry around the bismuth center with two mesityl substituents in the equatorial positions and two fluorine atoms of bridging (SbF_6_)^−^ counterions in the axial positions, leading to a one-dimensional coordination polymer in the solid state (Supporting Information). The central atom in [Bi­(Mes)_2_(thf)_2_(SbF_6_)] (**2**) interacts with two mesityl and two THF ligands, which also results in a bisphenoidal coordination geometry with the neutral ligands in the axial positions (Scheme 2b). The C–Bi–C angle of 108.9(3)° is significantly larger than that in related compounds such as [BiMe_2_(NC_5_H_5_)_2_]­[SbF_6_] (C–Bi–C, 92.3°)^58^ due to the steric demand of the mesityl substituents. The bond angles around the central atom that involve the THF ligands are close to the ideally expected values of 90° C–Bi–O (81.4(2)-94.3(2)°) and 180°, respectively, O–Bi–O (171.21(17)°).^58,53^ The closest Bi···F distance amounts to 3.53 Å, which is close to the sum of the van-der-Waals radii (3.54 Å),^59^ indicating that weak interactions should also be possible in the solid state. Comparing the relatively long Bi–C bonds in **2** (2.273(7) Å and 2.273(8) Å) to those of aryl bismuth cations without neutral ligands ([BiPh_2_(SbF_6_)]: 2.247(2)-2.257(2) Å, [BiDipp_2_(SbF_6_)] 2.257(2)-2.260(2) Å) or to similar compounds with a low steric profile of the hydrocarbon group ([BiMe_2_(NC_5_H_5_)_2_]­[SbF_6_] 2.235(12)-2.223(12) Å)^54,53,58^ underscores the impact of neutral ligands and the sterically demanding mesityl group.

With compounds [BiPh_2_(SbF_6_)] (**1**), [BiMes_2_(SbF_6_)­(thf)_2_] (**2**), and [BiDipp_2_(SbF_6_)­(tol)] (**3**) in hand, we set out to investigate the potential formation of Pt → Bi metal-only Lewis pairs. To this end, solutions of bismuth compounds **1**–**3** in toluene or difluorobenzene were added to solutions of Pt­(PCy_3_)_2_ in the same solvent. Upon addition, the color of the reaction mixtures turned from pale yellow to bright red (in the case of **1** and **2**) or red-orange to deep-red (in the case of **3**), but then changed to light orange over a period of two (for **1**) to 4 h (for **2** and **3**), while forming a black precipitate.

^31^P NMR spectroscopic reaction monitoring revealed the formation of a main compound (62% relative signal intensity) in the case of starting material **1** and exclusively one phosphorus-containing species in the case of **2** and **3**. No significant change in the spectra was observed over the course of the reaction (despite the precipitation of increasing amounts of a dark solid). The selective reactions of **2** and **3** were tested for solvent effects, but the rapid and selective formation of one new product was observed irrespective of the choice of solvent (toluene, 1,2-difluorobenzene, and THF).

The ^31^P NMR chemical shifts of the new compounds formed in reactions with **1**, **2**, and **3**, amount to 18.8, 27.0, and 31.2 ppm, respectively. This speaks against the formation of Pt → Bi Lewis pairs (δ­([(PCy_3_)_2_Pt→BiMe_2_(SbF_6_)]) = 53.4 ppm),^39^ but rather for the formation of Pt^II^ *bis*(phosphane) complexes, PtX_2_(PR_3_)_2_ (X = monoanionic ligand), in a square planar or “masked T-shaped”^60^ coordination geometry.^61–65^ The ^1^ *J*_PtP_ coupling constants (2796, 2801, and 2871 Hz) clearly favor a *trans*- over a *cis*-configuration.^62^ In the case of the less selective reaction (with starting material **1**) only trace amounts of product **4** could be obtained, which was sufficient for crystallographic analyses (*vide infra*). For the highly selective reactions (with starting materials **2** and **3**), products **5** and **6** could be isolated and fully characterized. Single-crystal X-ray diffraction experiments revealed the products **4**-**6** to show the composition [Pt­(PCy_3_)_2_(Ar)­(SbF_6_)] (Ar = Ph (**4**, triclinic space group *P*1̅, *Z* = 2), Mes (**5**, orthorhombic space group *Cmc2*_1_, Z = 4) or Dipp (**6**, monoclinic space group *P*2_1_/*c*, *Z* = 4); Figure 1). For **4** and **5** the platinum atom shows a square planar coordination geometry with two PCy_3_ ligands *trans* to each other, in agreement with the ^31^P NMR spectroscopic data. The aryl group and the (SbF_6_)^−^ unit occupy the remaining positions. For compound **6**, a T-shaped geometry is found due to the lack of a Pt···F contact *trans* to the Dipp substituent. This is ascribed to the higher steric demand of the Dipp group, which is supported by an analysis of the relevant bond lengths and angles. For instance, the P–Pt–P angle decreases in the order **4** (175.79(4)°) > **5** (169.68(8)°) > **6** (166.184(14)°) and the Pt–C bond lengths increase in the same order **4** (1.982(4) Å) < **5** (2.005(6) Å) < **6** (2.0081(15) Å). The Pt–P bond lengths are only marginally affected by the variations in steric bulk. Indications for agostic Pt···(H–C) interactions in the 14 valence-electron-compound **6** with its three-coordinate platinum center could not be deduced from structural parameters, since the shortest Pt···(*H*–C) and Pt···(H–*C*) distances amount to 2.4651–2.989 and 2.941(5)–3.187(3) Å, respectively. These interatomic distances are similar to those in a platinum boryl complex [Pt­(PCy_3_)_2_(BBrFc)]­[B­(3,5-(CF_3_)_2_-C_6_H_3_)_4_] (Fc = ferrocenyl), for which agostic interactions have been ruled out (Pt···H, 2.542 Å; Pt···C, 3.117 Å)^66^ and exceed those of compounds, for which agostic interactions have been reported (e.g.,: [PtMe­(P*i*Pr_3_)_2_]­[1-H-*closo*-CB_11_Me_11_] (Pt···H, 2.24 Å; Pt···C, 2.859 Å) and [Pt­(PCy_2_(2,6-Me_2_-C_6_H_3_))­(κ^2^-*P*,*C*-P­(2-Me-6-CH_2_C_6_H_3_)­Cy_2_)]­[B­(3,5-(CF_3_)_2_-C_6_H_3_)_4_] ((Pt···H, 2.057 Å; Pt···C, 2.432 Å))).^67,68^

**Figure 1:**
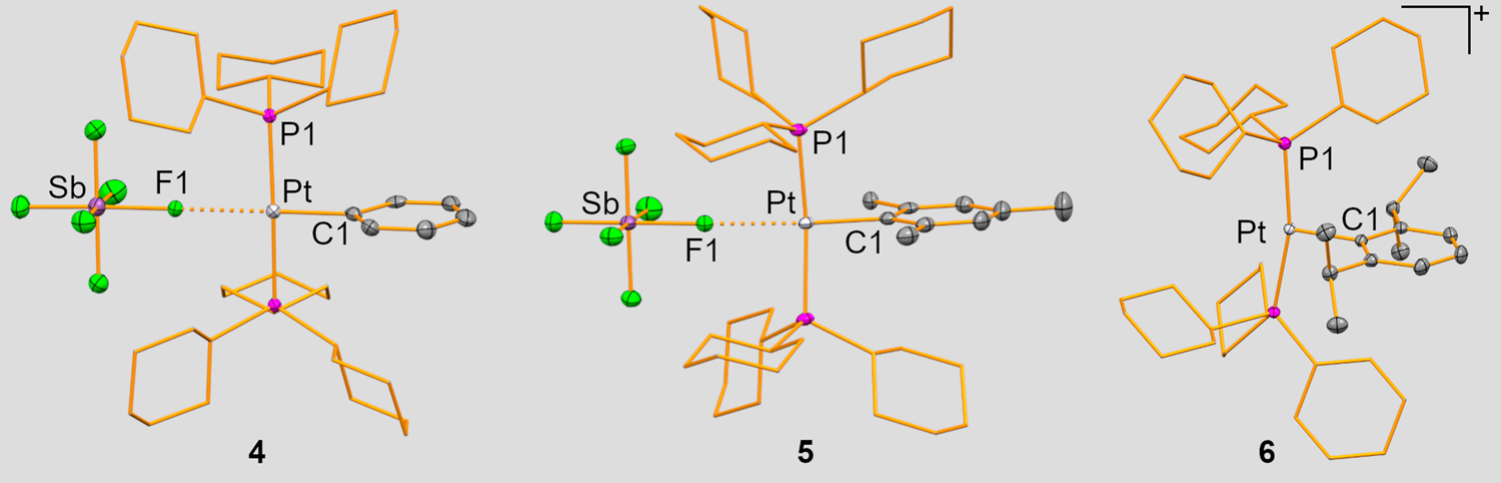
Molecular structures of **4**, **5**, and **6** in the solid state. Displacement ellipsoids are drawn at the 50% probability level. Hydrogen atoms, lattice-bound solvent molecules, and the (SbF_6_)^−^ counterion in **6** are omitted, and cyclohexyl groups shown as wireframe for clarity. Selected bond lengths (Å) and angles (deg) for **4**: Pt–C1 1.982(4), Pt–F1 2.419(2), Pt–P1 2.3420(10), P1–Pt–P2 175.79(4), C1–Pt–F1 179.62(14), C1–Pt–P1 91.42(12), F1–Pt–P1 88.91(6); for **5**: Pt–C1 2.005(6), Pt–F1 2.504(4), Pt–P1 2.3435(10), P1–Pt–P1′ 169.68(8), C1–Pt–F1 163.49(19), C1–Pt–P1 93.15(4), F1–Pt–P1 88.15(4); for **6**: Pt–C1 2.0081(15), Pt–P1 2.3531(4), P1–Pt–P2 166.184(14), C1–Pt–P1 96.79(4), Pt···F 5.9240(10).

In solution, compounds **4**–**6** do not show strong bonding interactions between the platinum atoms and the (SbF_6_)^−^ units, as indicated by ^19^F NMR spectroscopy. In the ^19^F NMR spectra, the distinct resonance for non**-** or very weakly coordinating (SbF_6_)^−^ was detected between −106 and −145 ppm. This NMR spectroscopic signal results from two overlaying multiplets, one sextet due to the ^1^ *J*_SbF_ coupling between ^19^F and ^121^Sb (natural abundancy 57%, *I* = 5/2) and one octet due to the ^1^ *J*_SbF_ coupling between ^19^F and ^123^Sb (natural abundancy 43%, *I* = 7/2). Only if a potential coordination between the central atom and its (SbF_6_)^−^ counteranion is weak or fluxional enough, a well-resolved resonance for an (SbF_6_)^−^ anion with apparent *O*_*h*_ symmetry will be obtained.^53^

The sequence of reactions that yields compounds **4**–**6** formally corresponds to the oxidative addition of the Bi–C bond of a bismuth complex cation [BiAr_2_(SbF_6_)] to a Pt^0^ center (Pt^0^ → Pt^II^), followed by a reductive elimination that yields low-valent bismuth compounds “BiR” (Bi^III^ → Bi^I^), which are subject to subsequent redox-disproportionation (3 Bi^I^ → Bi^III^ + 2 Bi^0^) (Scheme 3, bottom). This contrasts with the simple Lewis pair formation Pt­(PCy_3_)_2_ + BiR_2_(SbF_6_) → [(Cy_3_P)_2_Pt→BiR_2_]­[SbF_6_] that has been observed under identical conditions for the methyl analog (R = Me) (Scheme 3, top). Interestingly, the originally targeted adduct formation between Pt­(PCy_3_)_2_ and the model compound [BiPh_2_]^+^ to give [(PCy_3_)_2_Pt→BiPh_2_]^+^ is strongly exergonic according to DFT calculations (Δ*G* = −47.4 kcal·mol; Supporting Information). When analyzing subsequent reactions of compounds [(PCy_3_)_2_Pt→BiR_2_]^+^, however, oxidative aryl transfer from Bi to Pt (R = Ph, experimentally observed) is thermodynamically more favorable than the transfer of an alkyl group (R = Me, experimentally not observed). The calculations suggest the redox disproportionation of bismuthinides BiR to give BiR_3_ and Bi^0^ to be an important thermodynamic driving force of the reaction (Supporting Information). In order to experimentally evaluate the ability of different types of bismuth cations to be involved in the net oxidation of the platinum center, [BiMe_2_(SbF_6_)] and **1**–**3** were analyzed by cyclic voltammetry. Under reducing conditions, an irreversible electron transfer with a peak potential of −2.34 ([BiMe_2_(SbF_6_)]), −2.02 (**1**), −2.31 (**2**), −2.12 (**3**) V vs Fc/Fc^+^ was observed, indicating a cathodic shift by 30–320 mV for the aryl species compared to the methyl compound. This points toward the less electron-donating character of the aryl groups (as compared to methyl ligands) as a relevant factor in facilitating the sequence of reactions leading to compounds **4**–**6**. The reactions described herein are equivalent to a net oxidative aryl transfer from a bismuth center to a transition metal atom. While redox-neutral transmetalation events have been reported in a significant number of cases with a focus on Cu and Pd catalysis,^46,47,51,69^ the net oxidative transfer of a simple aryl ligand from a well-defined bismuth precursor to give an isolable bismuth-free transition metal complex adds a new facet to the reactivity between bismuth compounds and transition metal complexes.^70–73^

**Scheme 3:**
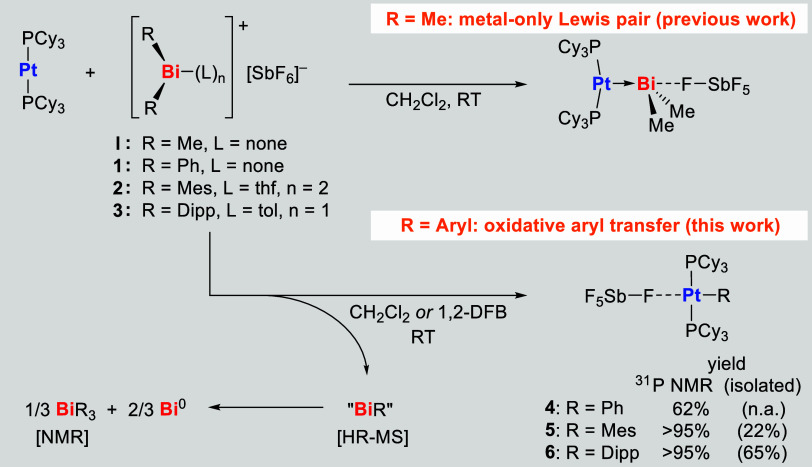
Reactions of **1**–**3** with Pt­(PCy_3_)_2_ to Give **4**–**6** ^a^

a 1,2-DFB = 1,2-difluorobenzene. Low isolated yields of **5** are due to difficulties in separating bismuth-containing byproducts of the reaction.

Importantly, the formation of **4**-**6** formally also generates low-valent bismuth compounds BiR as byproducts (Scheme 3, bottom). HRMS analyses of samples withdrawn at early stages of the reaction support the formation of these reactive intermediates, and ^1^H NMR spectroscopy indicated the formation of BiR_3_ at later stages of the reaction (in the case of R = Mes, Dipp), which is the expected product of the redox-disproportionation of BiR (according to 3 BiR → BiR_3_ + 2 Bi^0^).^74,75^

Attempts to trap the suggested reactive intermediate BiR have not been successful to date. Also in the presence of trapping reagents, compounds BiR_3_ have been detected by NMR spectroscopy and HRMS analyses as a result of redox disproportionation events (cf. Scheme 3, bottom left). Instead, trapping reactions with 3,5-di-*tert*-butyl-1,2-benzoquinone led to the formation of [Pt­(PCy_3_)_2_(O_2_-3,5-*t*Bu_2_-C_6_H_2_)]­[SbF_6_] (**7**) (Scheme 4a). This compound was identified through single crystal X-ray diffraction analysis (monoclinic space group *P*2_1_/*n*, Z = 4; Scheme 4b) and shows a distorted square planar coordination geometry around the platinum center. The chelating nature of the oxygen-based ligand enforces a small bite angle and a *cis* arrangement (O–Pt–O, 78.48(13)°), leading to a larger P–Pt–P angle of 105.89(4)°. The (SbF_6_)^−^ anion does not show directional bonding interactions with the platinum center, as judged by distance criteria. The Pt–P bond lengths do not differ significantly (2.2633(12)–2.2666(13) Å) and are very close to those reported for the Pt^II^ catecholate complex [Pt­(PCy_3_)_2_(O_2_C_6_H_4_)] (**I**) (Pt–P, 2.266(2)-2.268(2) Å).^76^ In contrast, the Pt–O distances show considerable variations (2.060(3)-2.109(3) Å) and are on average significantly longer than those in Pt^II^ catecholates such as **I** (Pt–O, 2.033(5)-2.051(5) Å) or [Pt­(dppe)­(O_2_C_6_H_4_)] (**II**) (Pt–O, 2.039(3)-2.056(3) Å; dppe = Ph_2_PC_2_H_4_PPh_2_).^76,77^ In turn, the C–O bond lengths in **7** (1.301(6)-1.281(6) Å) are between those in Pt^II^ catecholates (**I**: C–O, 1.338–1.347 Å; **II**: C–O, 1.340(5)-1.370(5) Å) and those in the free quinone O_2_-3,5-*t*Bu_2_-C_6_H_2_ (C–O, 1.214(3)–1.217(3) Å).^78^ Altogether, this points toward the formation of a cationic Pt^II^ species coordinated by a radical semiquinolate ligand (O_2_-3,5-*t*Bu_2_-C_6_H_2_)^•^. In agreement with this, compound **7** was NMR silent. Its composition was confirmed by HRMS analysis, with *m*/*z* = 975.573, corresponding to the molecular ion peak, and supported by its rational synthesis from Pt­(PCy_3_)_2_, 3,5-di-*tert*-butyl-1,2-benzoquinone, and AgSbF_6_ (see Experimental Section). To probe the radical nature of **7**, EPR spectroscopic experiments with solutions of isolated **7** were performed. Indeed, a broad resonance was detected, unambiguously confirming the radical character of compound **7** (Scheme 4d). The *g*_iso_ value of 2.0024 is in agreement with values reported for related compounds that have been generated in situ.^79^ The broadness of the resonance indicates coupling of the unpaired spin with multiple coupling partners. This was confirmed by DFT calculations at the B3LYP-D3/def2-TZVP level of theory, which indicate the spin density to be delocalized through the semiquinolate ligand, with additional low spin density being found at the P and Pt atoms (Scheme 4c). With slight modifications of the instrumental parameters of the EPR spectrometer, the presence of hyperfine interactions could be confirmed. However, only a moderate resolution could be obtained due to the presence of multiple coupling partners so that the assignment of coupling constants has to be taken as a tentative suggestion (Supporting Information). While the synthesis and isolation of diamagnetic platinum catecholates have previously been achieved,^76,77,80,81^ reports on the more challenging-to-handle platinum semiquinolates are rare.^79^ To the best of our knowledge, such species have only been generated *in situ* so far, followed by spectro-electrochemical characterization;^79^ thus, compound **7** represents the first example of an isolated platinum semiquinolate complex, suggesting further explorations of this class of platinum compounds with redox-active ligands should be possible.

**Scheme 4:**
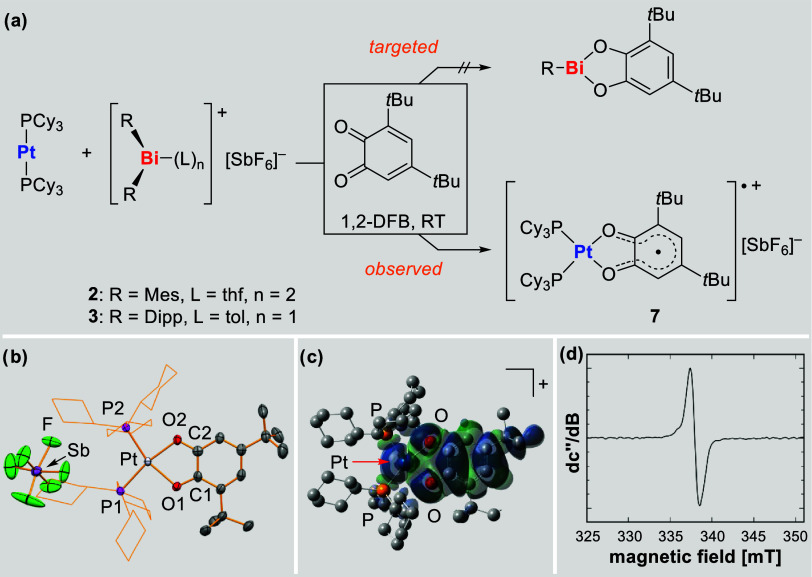
(a) Reaction of Pt­(PCy_3_)_2_ with **2** or **3** and O_2_-3,5-*t*Bu_2_-C_6_H_2_ to Give **7** (1,2-DFB = 1,2-Difluorobenzene) Instead of the Desired Bismuth Catecholate. (b) Molecular Structure of **7** in the Solid State. Displacement Ellipsoids are Drawn at 50% Probability Level, Hydrogen Atoms and Lattice-Bound Solvent Molecules are Omitted, Cyclohexyl Groups Shown as Wireframe for Clarity. Selected Bond Lengths [Å] and angles [deg]: Pt–P1 2.2666(13), Pt–P2 2.2633(12), Pt–O1 2.109(3), Pt–O2 2.060(3), O1–C1 1.301(6), O2–C2 1.281(6), P1–Pt–P2 105.89(4), O1–Pt–O2 18.48(13), O1–Pt–P1 91.03(10), O1–Pt–P2 162.98(10). (c) Spin Density Distribution of [Pt­(PCy_3_)_2_(O_2_-3,5-*t*Bu_2_-C_6_H_2_)]^+^ (**7** ^**+**^) as Determined by DFT Calculations (Isovalue = 0.0001; H Atoms Omitted for Clarity). (d) Continuous-Wave X-Band EPR Spectrum of a THF Solution of **7** (c = 4·10^–4^ mol/L). The Observed Resonance Shows a *g*_iso_ Value of 2.0024. Spectrometer Settings: Microwave Frequency = 9.473621 GHz, 0.02 mT Modulation Amplitude at 100 kHz, Microwave Power = 1.0 mW, Number of Accumulated Scans = 1, Conversion Time = 1 ms

## Conclusions

In conclusion, we have investigated a small series of simple diaryl bismuth cations [BiR_2_(L)*_n_*]^+^ (R = Ph, Mes, or Dipp; L = neutral ligand, *n* = 0–2) in the context of metal-only Lewis pair formation. This includes a new member that has been added to this series of rare compounds (R = Mes). Surprisingly, the aryl species [BiR_2_(L)*_n_*]^+^ do not undergo simple Lewis pair formation with the electron-rich platinum compound Pt­(PCy_3_)_2_, which is in stark contrast to the behavior of the alkyl complex cation [BiMe_2_]^+^. The diaryl bismuth cations undergo a sequence of reactions, resulting in the net oxidative aryl transfer to give [PtR­(PCy_3_)_2_(SbF_6_)]. This adds a new facet to the interaction of bismuth compounds with transition metal complexes, for which Lewis pair formation, oxidative addition reactions, and redox-neutral aryl transfer (i.e., ligand exchange reactions) have previously been reported. The bismuth-containing products of these reactions are suggested to be short-lived bismuthinidenes, BiR, which is supported by in situ mass spectrometry and the analysis of follow-up products. Attempted trapping reactions unexpectedly gave the first example of an isolable cationic platinum­(II) complex featuring a semiquinolate radical ligand.

## Experimental Section

All experiments were conducted under an atmosphere of dry argon using Schlenk and glovebox techniques. Solvents were degassed and purified according to standard laboratory procedures. NMR spectra were recorded on Bruker Avance spectrometers operating at 300 or 500 MHz with respect to ^1^H. ^1^H and ^13^C NMR chemical shifts are reported relative to SiMe_4_ using the residual signal of the deuterated solvent as a secondary standard.^57^ The assignment of resonances in ^1^H and ^13^C NMR spectra has been underlined by 2D experiments, such as HSQC and HMBC NMR spectroscopy. ^19^F and ^31^P NMR chemical shifts are reported relative to CFCl_3_ or 85% aqueous H_3_PO_4_, respectively, as external standards. Mass spectrometry was conducted on a Thermo Fischer Scientific Orbitrap Q Exactive Plus using ESI as an ionization method. The samples were infused into the mass spectrometer under an inert atmosphere through a syringe pump. Elemental analyses (C, H, N) were performed on a vario MICRO cube. Cyclic voltammograms were recorded by using a Gamry Interface 1010 potentiostat and a three-electrode setup and a concentration of 0.1 mol/L NBu_4_PF_6_ as a conductive salt. EPR spectra were recorded on a Bruker Magnettech ESR5000 spectrometer operating in the X-Band (9.4 GHz). Samples were prepared in an argon-filled glovebox and transferred into a quartz glass tube prior to data collection. All measurements were performed under an atmosphere of purified argon. Simulations of the obtained EPR spectra were done with the EasySpin software package,^16^ running in the MATLAB software environment.^82^ Single-crystals suitable for X-ray diffraction were coated with polyisobutylene or perfluorinated polyether oil in a glovebox, transferred to a nylon loop, and then transferred to the goniometer of a Bruker D8 Quest or D8 Venture diffractometer equipped with a molybdenum (λ = 0.71073 Å) X-ray tube. Using Olex2,^83^ the structures were solved with the XT structure solution program^84^ using intrinsic phasing and refined with the XL refinement package using least-squares minimization.^85^ All non-hydrogen atoms were refined anisotropically. Hydrogen atoms were included in the structure factor calculations. All hydrogen atoms were assigned to idealized geometric positions. Deposition numbers 2500345–2500349 contain the supplementary crystallographic data for this paper. These data are provided free of charge by the joint Cambridge Crystallographic Data Centre and Fachinformationszentrum Karlsruhe Access Structures service www.ccdc.cam.ac.uk/structures.

### Preparation of [BiMes_2_(SbF_6_)­(thf)_2_] (**2**)

BiMes_2_Cl (50 mg, 0.104 mmol, 1.0 equiv) was dissolved in THF (2 mL) and combined with a solution of AgSbF_6_ (36 mg, 0.104 mmol, 1.0 equiv) in THF (3 mL). Upon addition, the solution turns bright yellow, and a colorless precipitate is formed. After filtration and extracting the precipitate with THF (2 mL), the THF phases were combined and stored at −30 °C. After 20 h needle-like crystals of [BiMes_2_(SbF_6_)­(thf)_2_] had formed that were isolated by filtration and dried *in vacuo* (50 mg, 0.60 mmol, 58%). ^1^H NMR (500 MHz, CD_2_Cl_2_) δ = 1.84 (m, 8 H, thf), 2.30 (s, 6 H, *para*–C*H*_3_), 2.43 (s, 12 H, *ortho*–C*H*_3_), 3.71 (m, 8 H, thf), 7.59 (s, 4 H, *meta*–C*H*) ppm. ^13^C­{^1^H}-NMR (125 MHz, CD_2_Cl_2_): δ = 21.74 (s, *para*-*C*H_3_), 25.99 (s, thf), 26.70 (s, *ortho*-*C*H_3_), 70.51 (s, thf), 133.82 (s, *meta*-*C*H), 141.53 (s, *para*-*C*), 147.06 (s, *ortho*-*C*), 206.94 (s, *ipso*-*C*) ppm. ^19^F-NMR (283 MHz, CD_2_Cl_2_): δ = −124.0 (br, Sb*F*_6_) ppm. Elemental analysis: calcd for [C_26_H_38_BiF_6_O_2_Sb] (827.31 g/mol): C 37.75, H 4.63; found: C 37.39, H 4.71.

Attempts to prepare BiMes_2_(SbF_6_) (i.e., free of neutral ligands) were unsuccessful to date, presumably due to the poor solubility of this species in weakly coordinating solvents such as dichloromethane and 1,2-difluorobenzene.

### Attempted Preparation of [Pt­(PCy_3_)_2_(Ph)­(SbF_6_)] (**4**)

[Bi­(Ph)_2_(SbF_6_)] (16 mg, 0.026 mmol, 1.0 equiv) was dissolved in 1,2-difluorobenzene (2 mL) and added to a solution of Pt­(PCy_3_)_2_ (20 mg, 0.026 mmol, 1.0 equiv) in 1,2-difluorobenzene (3 mL). The reaction mixture turned bright red at the beginning and yellowish brown after a few seconds. After 2 h, a dark precipitate had formed. The solution was filtered, dried *in vacuo* and redissolved in dichloromethane. Layering this solution with *n*-pentane (5 mL) at −30 °C led to the formation of a small amount of yellow crystals after 4 d that allowed the unambiguous confirmation of the connectivity in **4** by single-crystal X-ray analysis. All attempts to isolate **4** in larger amounts have been unsuccessful to date due to the presence of inseparable byproducts.

^19^F-NMR (283 MHz, CD_2_Cl_2_): δ = −106 bis −145 (br, m, Sb*F*_6_) ppm. ^31^P­{^1^H}-NMR (122 MHz, CD_2_Cl_2_): δ = 18.82 (s, ^1^ *J*_PPt_ = 2796 Hz) ppm. HRMS (ESI): calcd for [C_42_H_71_P_2_Pt]^+^: *m*/*z* = 832.4677, found: *m*/*z* = 832.4660; calculated for [C_18_H_33_P] + H^+^: *m*/*z* = 281.2393, found: 281.2387.

### Preparation of [Pt­(PCy_3_)_2_(Mes)­(SbF_6_)] (**5**)

[BiMes_2_(SbF_6_)­(thf)_2_] (50 mg, 0.06 mmol, 1 equiv) was dissolved in toluene and added to a solution of Pt­(Pcy_3_)_2_ (46 mg, 0.06 mmol, 1 equiv) in toluene. The dark orange reaction mixture was stirred for 4 h. During this time, the color brightened up and a dark precipitate was formed. After filtration, all volatiles were removed under reduced pressure and the remaining solid was washed with pentane (3 × 2 mL) and extracted with a mixture of dichloromethane and *n*-pentane (1:3, 2 × 2 mL). Slow evaporation of the solvent mixture at −30 °C led to the formation of orange crystals of [Pt­(PCy_3_)_2_(Mes)­(SbF_6_)], which were isolated by filtration and dried *in vacuo* (15 mg, 0.013 mmol, 22%).

^1^H NMR (500 MHz, CD_2_Cl_2_) δ = 1.19–1.26 (br, m, 18 H, overlap of 12 H of 2,6-PCy_3_, and 6 H of 4-PCy_3_), 1.34–1.42 (br, m, 12 H, 3,5-PCy_3_) 1.72–1.82 (br, m, 30 H, overlap of 12 H of 2,6-PCy_3_, 12 H of 3,5-PCy_3_ and 6 H of 4-PCy_3_), 2.18 (s, 3 H, Mes-*para*–C*H*_3_), 2.21–2.28 (br, m, 6 H, 1-PCy_3_), 2.71 (s, 6 H, Mes-*ortho*–C*H*_3_), 6.49 (s, 2 H, Mes-*meta*–C*H*) ppm. ^13^C­{^1^H}-NMR (126 MHz, CD_2_Cl_2_) δ = 19.95 (s, Mes *para*-*C*H_3_, detected via ^1^H–^13^C-HSQC spectrum), 26.32 (s, 4-PCy_3_), 27.84 (vt, ^2^ *J*_PC_ = 5.3 Hz, 2,6-PCy_3_), 28.46 (s, Mes *ortho*-*C*H_3_, detected via ^1^H–^13^C-HSQC spectrum) 30.66 (s, 3,5-PCy_3_), 35.57 (vt, ^1^ *J*_PC_ = 12.7 Hz, 1-PCy_3_), 121.73 (s, Mes *ortho*-*C*, detected via ^1^H–^13^C-HMBC spectrum), 127.74 (s, Mes *meta*-*C*H), 134.65 (s, Mes *ipso*-*C*, detected via ^1^H–^13^C-HMBC spectrum), 134.69 (s, Mes *para*-*C*, detected via ^1^H–^13^C-HMBC spectrum) ppm. ^19^F-NMR (283 MHz, CD_2_Cl_2_): δ = −106 bis −145 (br, m, Sb*F*_6_) ppm. ^31^P­{^1^H}-NMR (122 MHz, CD_2_Cl_2_): δ = 27.01 (s, ^1^ *J*_PPt_ = 2801 Hz) ppm. HRMS (ESI): calculated for [C_45_H_77_P_2_Pt]^+^: *m*/*z* = 874.5147, found: *m*/*z* = 874.5120; calculated for [C_18_H_33_P] + H^+^: *m*/*z* = 281.2393, found: 281.2384.

### Preparation of [Pt­(PCy_3_)_2_(Dipp)­(SbF_6_)] (**6**)

[Bi­(Dipp)_2_(SbF_6_)­(tol)] (60 mg, 0.070 mmol, 1 equiv) was dissolved in 1,2-difluorobenzene (3 mL) and added to a solution of Pt­(PCy_3_)_2_ (53 mg, 0.070 mmol, 1 equiv) in 1,2-difluorobenzene (4 mL), leading to a darkening of the red color. The reaction mixture was thoroughly stirred for 4 h at room temperature. During this time, the color brightened up, and a dark precipitate was formed. All volatiles were removed under reduced pressure. The crude product was dissolved in dichloromethane (1 mL) and filtered. The filtrate was layered with a mixture of diethyl ether (3 mL) and *n*-pentane (5 mL). After 5 days, red crystals of [Pt­(PCy_3_)_2_(Dipp)­(SbF_6_)] had formed, were isolated by filtration, and dried *in vacuo* (49 mg, 0.042 mmol, 61%).

^1^H NMR (500 MHz, CD_2_Cl_2_) δ = 1.18–1.50 (br, m, 30 H, overlap of 12 H of 2,6-PCy_3_, 12 H of 3,5-PCy_3_ and 6 H of 4-PCy_3_), 1.37 (d, 12 H, ^3^ *J*_HH_ = 6.7 Hz, Dipp *iso*-propyl–C*H*_3_, in overlap with the PCy_3_ signals), 1.55–2.04 (br, m, 30 H, overlap of 12H of 2,6-PCy_3_, 12 H of 3,5-PCy_3_ and 6 H of 4-PCy_3_), 2.23–2.52 (br, s, 6 H, 1-PCy_3_), 4.18 (sept, 2 H, ^3^ *J*_HH_ = 6.7 Hz, Dipp *iso*-propyl–C*H*), 6.60 (d, 2 H, ^3^ *J*_HH_ = 7.4 Hz, Dipp *meta* C*H*), 6.94 (t, 1 H, ^3^ *J*_HH_ = 7.4 Hz, Dipp *para* C*H*) ppm. ^13^C­{^1^H}-NMR (126 MHz, CD_2_Cl_2_) δ = 25.52 (s, Dipp *iso*-propyl-*C*H_3_), 26.22 (s, 4-PCy_3_), 27.61 (t, ^2^J_PC_ = 5.2 Hz, 2,6-PCy_3_), 31.25 (br, s, 3,5-PCy_3_), 34.41 (br, t, ^1^J_PC_ = 11.4 Hz, 1-PCy_3_), 40.13 (s, Dipp *iso*-propyl-*C*H), 124.01 (s, Dipp *meta-C*H), 124.83 (s, Dipp *ortho-C*), 127.94 (s, Dipp *para-C*H), 144.26 (s, Dipp *ipso*-*C*) ppm. ^19^F-NMR (283 MHz, CD_2_Cl_2_): δ = −106 bis −143 (br, m, Sb*F*_6_) ppm. ^31^P­{^1^H}-NMR (122 MHz, CD_2_Cl_2_): δ = 31.21 (s, ^1^ *J*_PPt_ = 2871 Hz) ppm. Elemental analysis: calcd for [C_48_H_83_F_6_P_2_PtSb] (1152.97 g/mol): C 50.00, H 7.26; found: C 50.33, H 7.17.

### Preparation of [Pt­(PCy_3_)_2_(O_2_-3,5-*t*Bu_2_-C_6_H_2_)­(SbF_6_)] (**7**)

Pt­(PCy_3_)_2_ (60 mg, 0.079 mmol, 1 equiv) was dissolved in THF (3 mL) and added to a solution of 3,5-di-*tert*-butyl-1,2-benzoquinone (17 mg, 0.079 mmol, 1 equiv) in THF (1 mL). Upon addition, a yellow solution was obtained, to which AgSbF_6_ (30 mg, 0.079 mmol, 1 equiv) was added. The formation of a black precipitate was observed, which was filtered off after 5 min. The remaining dark greenish solution was layered with *n*-pentane and stored at −30 °C to form colorless crystals of [Pt­(PCy_3_)_2_(O_2_-3,5-*t*Bu_2_-C_6_H_2_)­(SbF_6_)] that were isolated by filtration and dried *in vacuo* (52 mg, 0.043 mmol, 54%). Due to the paramagnetic nature of the compound, no resonances could be detected via NMR spectroscopy. EPR spectra are presented and discussed in the main part and in the Supporting Information. HRMS (ESI): calcd for [C_50_H_86_O_2_P_2_Pt]^+^: *m*/*z* = 975.5750, found: *m*/*z* = 975.5734.

## Supplementary Material





## Notes

NMR Spectra, mass spectra, EPR spectroscopic, crystallographic and computational details, cyclic voltammograms, experimental details of trapping reactions (PDF)
Cartesian coordinates of DFT-geometry-optimized structures (XYZ)

## Abbreviations

Mes: 2,4,6-trimethyl-phenyl
Dipp: 2,6-di-*iso*-propyl-phenyl
Ph: phenyl
NMR: nuclear magnetic resonance
EPR: electron paramagnetic resonance
1,2-DFB: 1,2-difluorobenzene
