# Supplementary material for: Reactivity of Diaryl Bismuth Cations toward a Platinum(0) Complex: Oxidative Aryl Transfer
Source: Organometallics. 2026 Jan 6;45(2):188–96. doi: 10.1021/acs.organomet.5c00441 (PMC12848974; doi:10.1021/acs.organomet.5c00441)
Supplement: Supplementary file 1 [file om5c00441_si_001.pdf]

Supporting Information

Reactivity of Diaryl Bismuth Cations toward a Platinum(0) Complex: Oxidative Aryl Transfer

*Johannes Schwarzmann, Cissie Slopianka, and Crispin Lichtenberg**

Department of Chemistry, Philipps University Marburg, Hans-Meerwein-Straße 4,

35043 Marburg, Germany

Email: crispin.lichtenberg@chemie.uni-marburg.de

Table of Contents

1. NMR spectra 2
2. EPR spectroscopy 9
3. High resolution mass spectra 10
4. Cyclic voltammetry 14
5. Single-crystal X-ray diffraction analyses 19
6. Attempted trapping reactions (to trap suggested BiR intermediates) 24
7. Computational details 25
8. References 28

1. NMR spectra

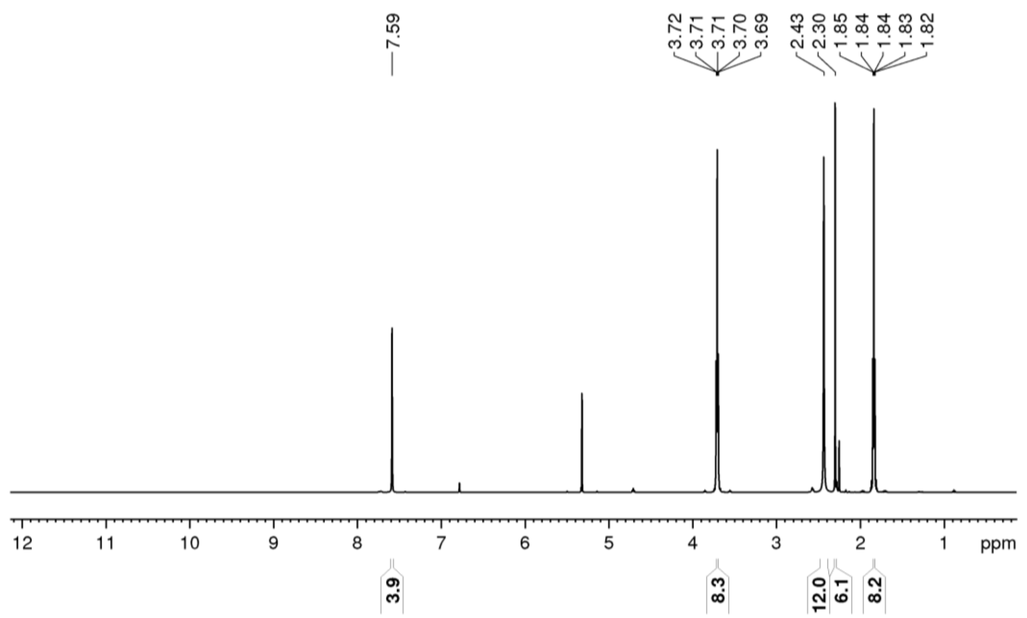

Figure S1. ^{1}H-NMR spectrum of [BiMes$_2$(SbF$_6$)(thf)$_2$] (**2**) in CD_2Cl_2.

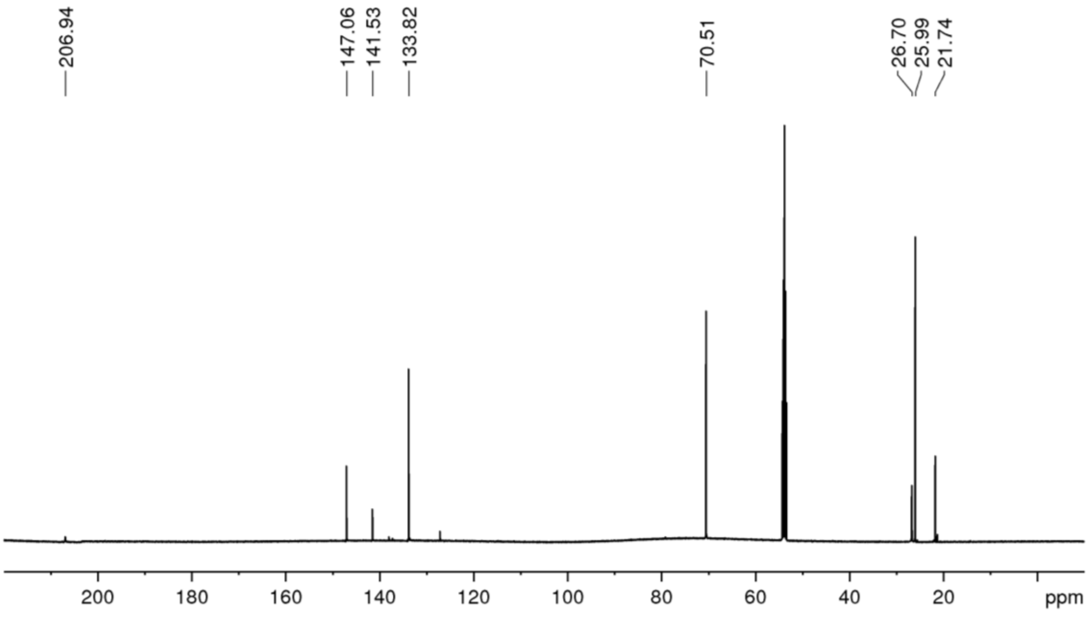

Figure S2. ^{13}C-NMR spectrum of [BiMes$_2$(SbF$_6$)(thf)$_2$] (**2**) in CD_2Cl_2.

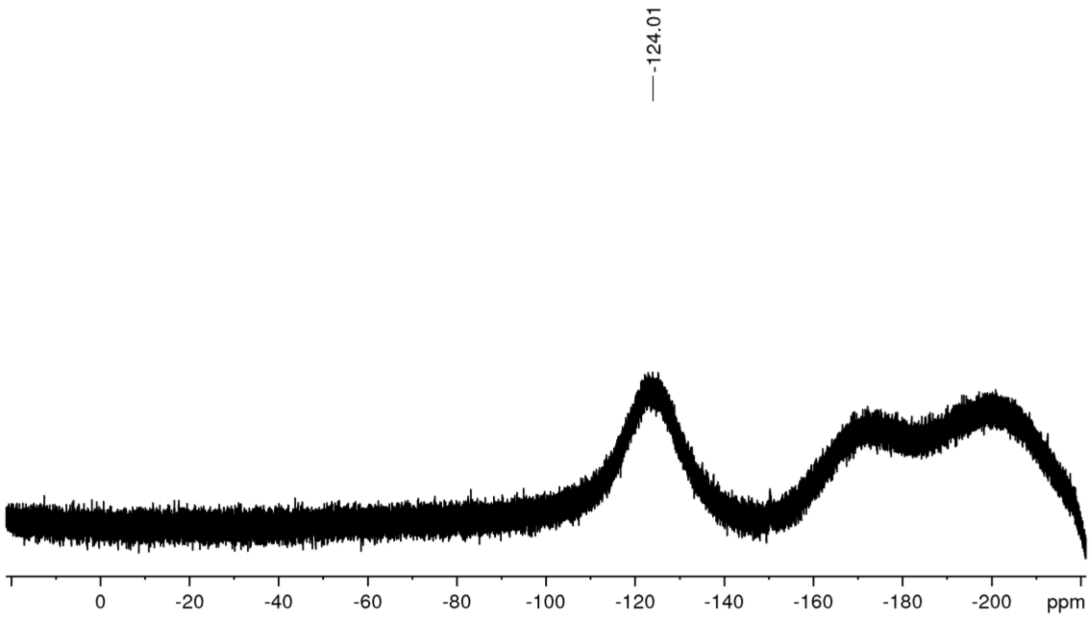

Figure S3. ^{19}F-NMR spectrum of [BiMes$_2$(SbF$_6$)(thf)$_2$] (**2**) in CD_2Cl_2.

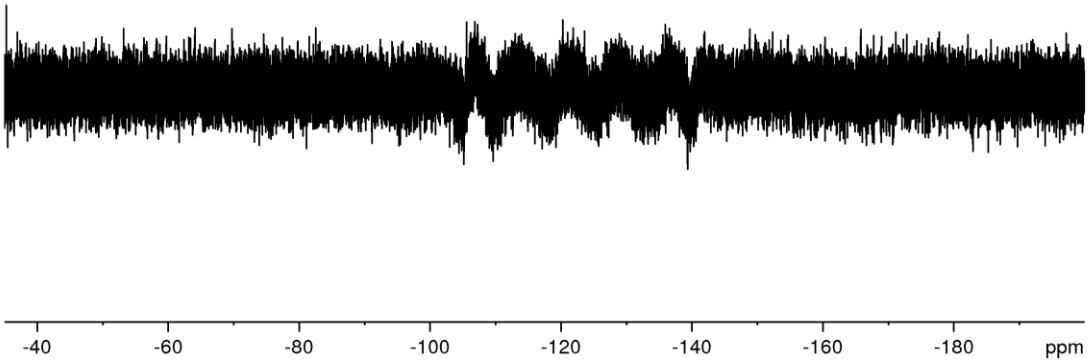

Figure S4. *in situ* ^{19}F-NMR spectrum of [Pt(PCy$_3$)$_2$Ph(SbF$_6$)] (**4**) in CD_2Cl_2.

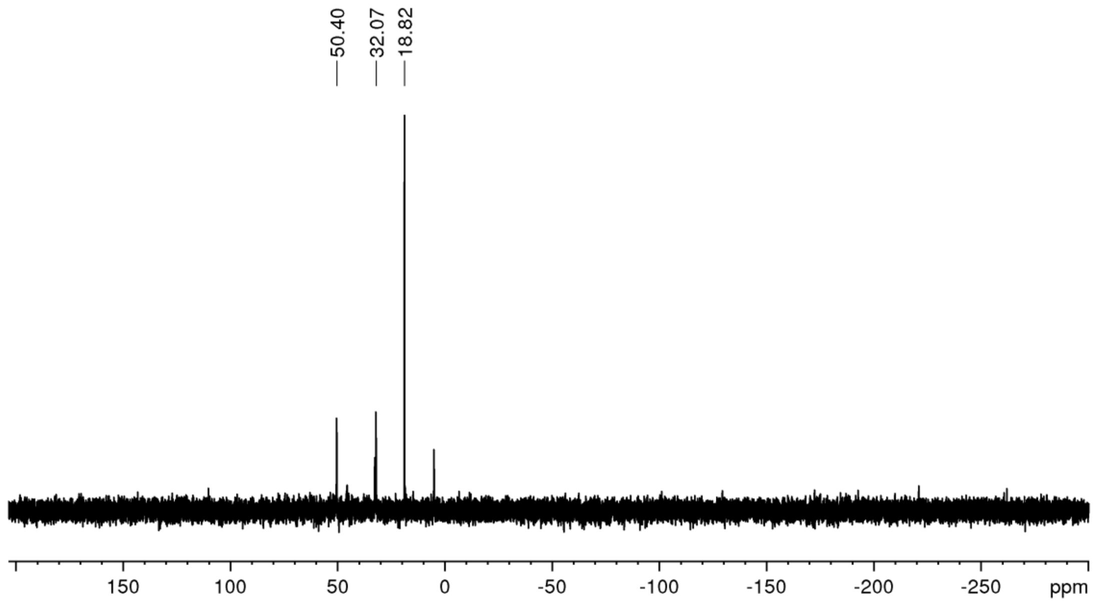

Figure S5. *in situ* ^{31}P-NMR spectrum of [Pt(PCy$_3$)$_2$Ph(SbF$_6$)] (**4**) in CD$_2$Cl$_2$.

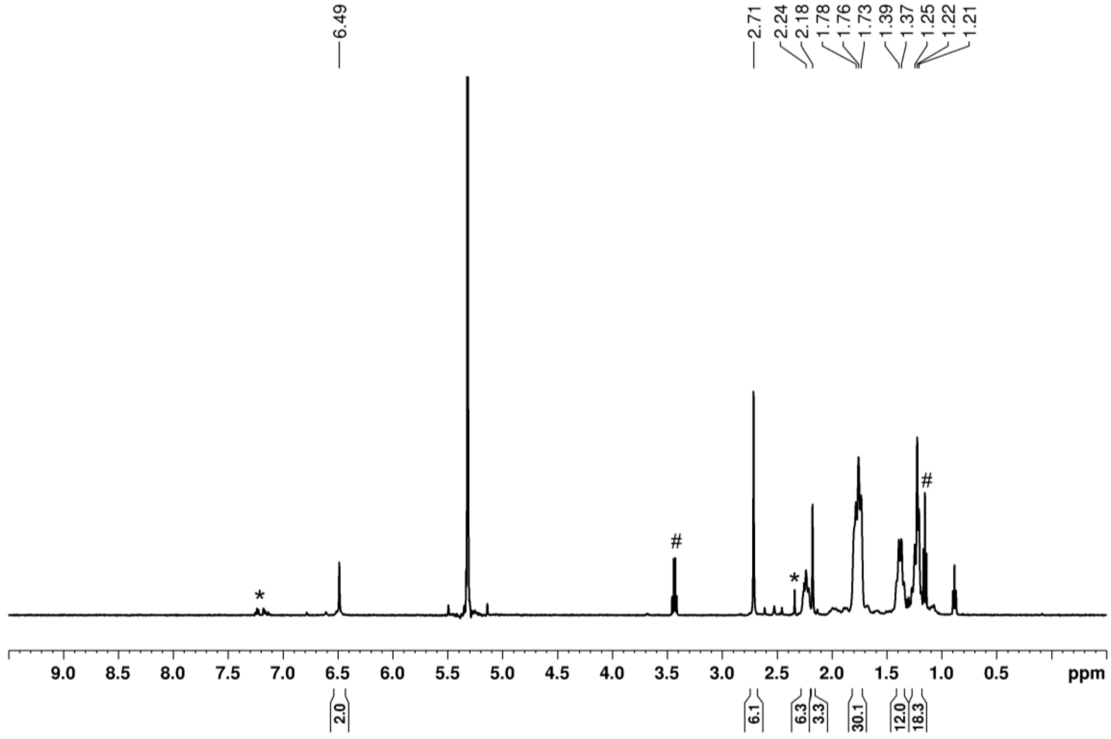

Figure S6. ^{1}H-NMR spectrum of [Pt(PCy$_3$)$_2$Mes(SbF$_6$)] (**5**) in CD$_2$Cl$_2$ (* = residual toluene, # = residual diethylether).

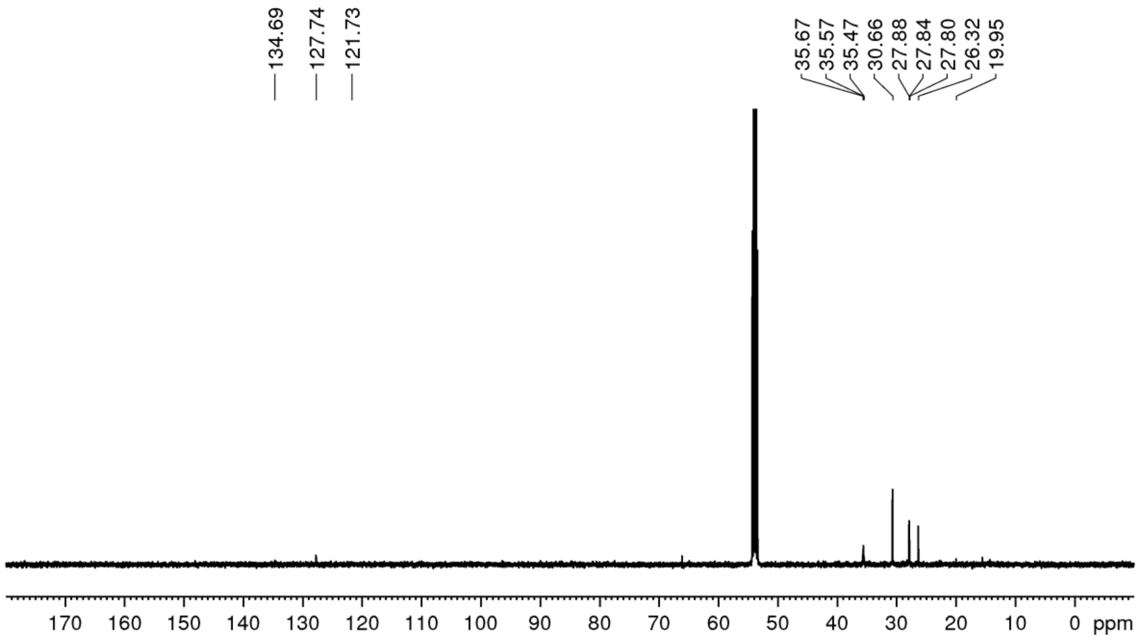

Figure S7. ^{13}C-NMR spectrum of $[Pt(PCy_3)_2Mes(SbF_6)]$ (**5**) in CD_2Cl_2.

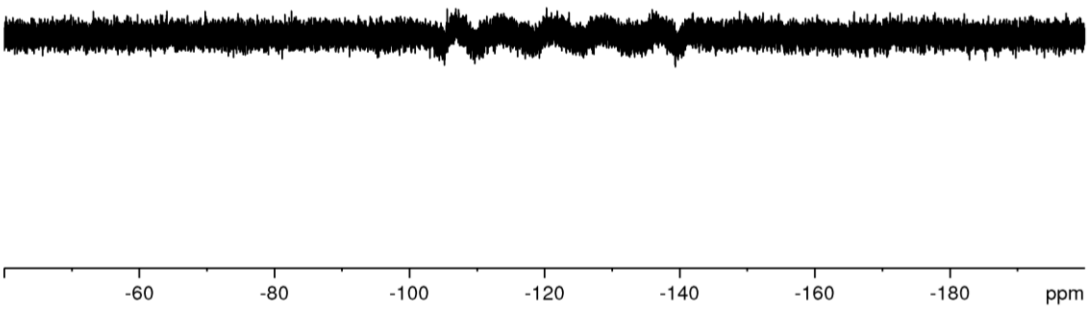

Figure S8. ^{19}F-NMR spectrum of $[Pt(PCy_3)_2Mes(SbF_6)]$ (**5**) in CD_2Cl_2.

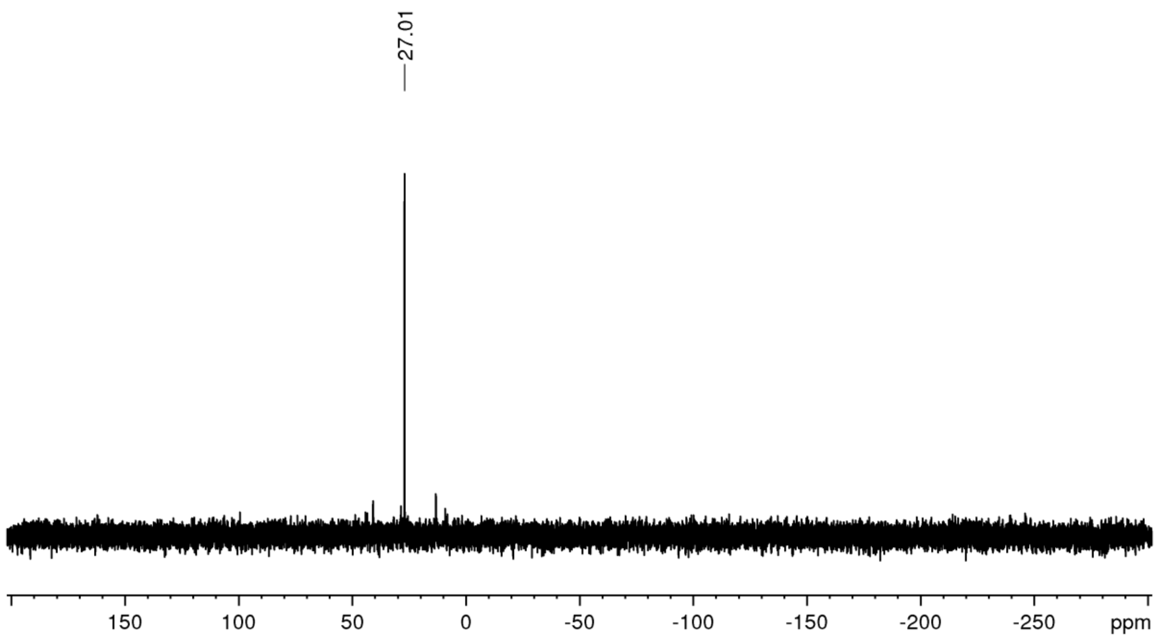

Figure S9. ^{31}P-NMR spectrum of [Pt(PCy_3)$_2$Mes(SbF_6)] (**5**) in CD_2Cl_2.

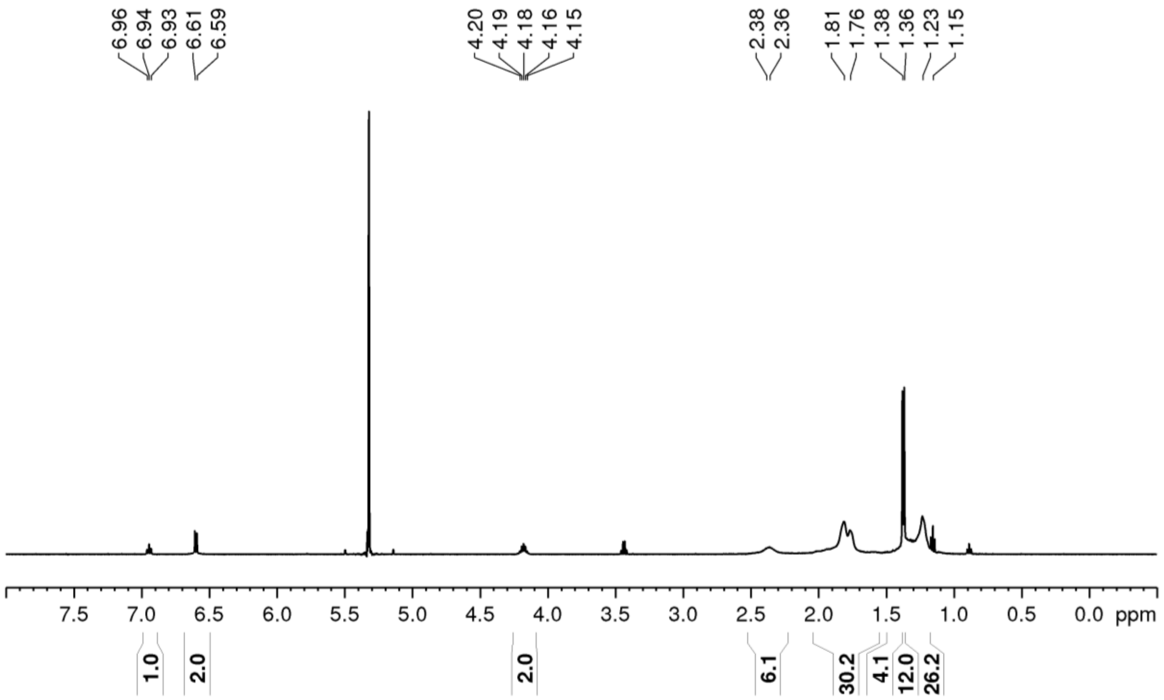

Figure S10. ^{1}H-NMR spectrum of [Pt(PCy_3)$_2$Dipp(SbF_6)] (**6**) in CD_2Cl_2.

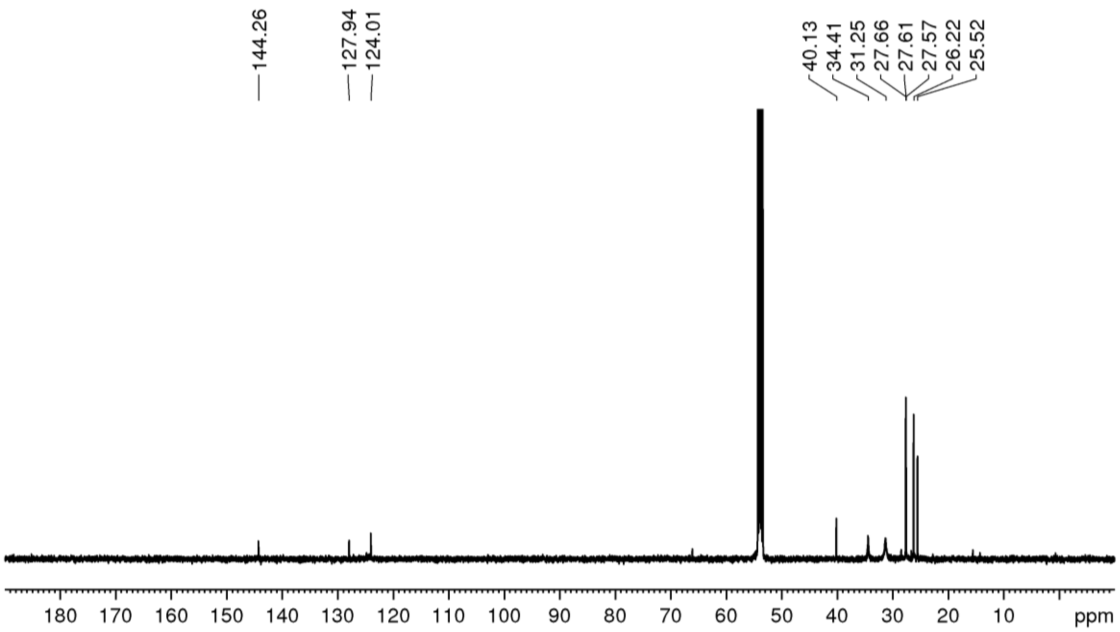

Figure S11. ^{13}C-NMR spectrum of [Pt(PCy$_3$)$_2$Dipp(SbF$_6$)] (**6**) in CD_2Cl_2.

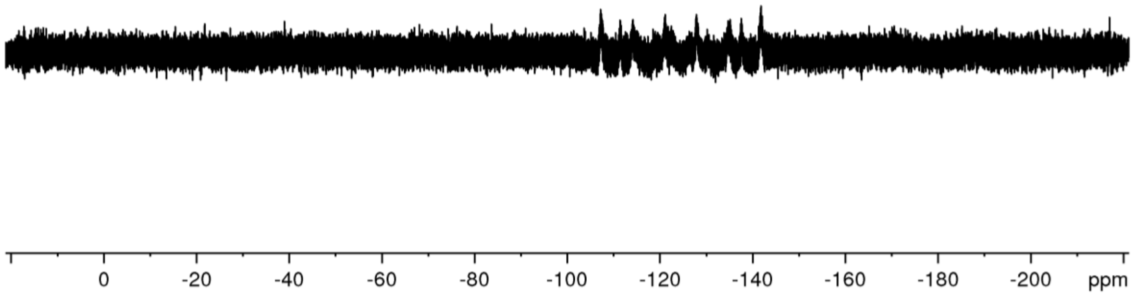

Figure S12. ^{19}F-NMR spectrum of [Pt(PCy$_3$)$_2$Dipp(SbF$_6$)] (**6**) in CD_2Cl_2.

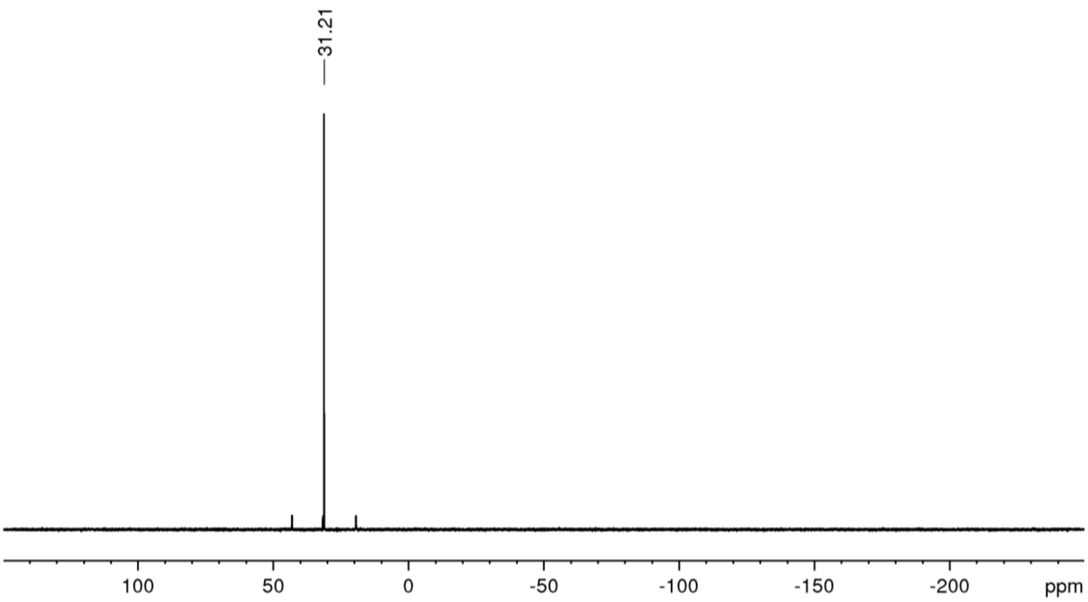

Figure S13. ^{31}P-NMR spectrum of [Pt(PCy_3)$_2$Dipp(SbF_6)] (**6**) in CD_2Cl_2.

2. EPR spectroscopy

EPR spectroscopic analyses of **7** are described in the main part. With modified instrumental parameters, hyperfine couplings could be detected. However, these are not well-resolved and a large number of coupling partners has to be considered so that the coupling constants obtained from a theoretical fit of the data have to be taken as a tentative suggestion.

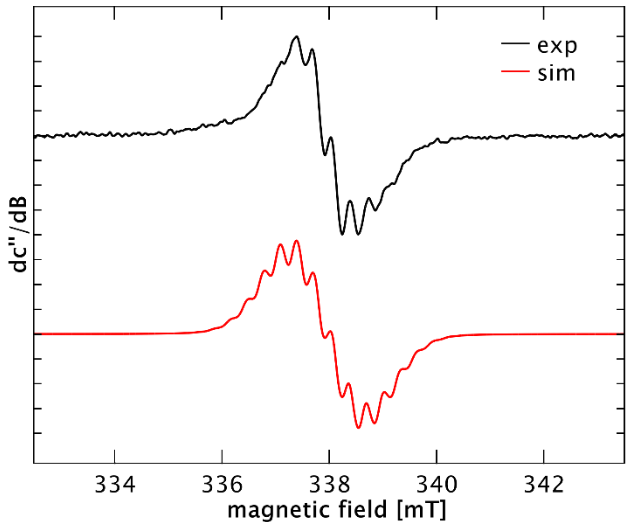

Figure S14. Experimental (black) and simulated (red) continuous-wave (CW) X-band EPR spectra of a THF solution of $[Pt(PCy_3)_2(O_2\text{-}3,5\text{-}t Bu_2\text{-}C_6H_2)][SbF_6]$ ($c = 4 \cdot 10^{-4}$ mol/L) at 25 °C. The observed resonance may be associated with the following coupling constants (see text): a(1 × ^{1}H) = 6.87 MHz (2.45 G, 0.245 mT), a(1 × ^{1}H) = 3.99 MHz (1.42 G, 0.142 mT), a(9 × ^{1}H) = 7.94 MHz (2.83 G, 0.283 mT), a(9 × ^{1}H) = 9.01 MHz (3.21 G, 0.321 mT), a(1 × ^{195}Pt) = 1.15 MHz (0.410 G, 0.0410 mT), a(1 × ^{31}P) = 2.66 MHz (0.949 G, 0.0949 mT), a(1 × ^{31}P) = 1.43 MHz (0.510 G, 0.0510 mT) and a g_{iso} value of 2.0024. Spectrometer settings: microwave frequency = 9.473651 GHz, 0.01 mT modulation amplitude at 100 kHz, microwave power = 1.0 mW, number of accumulated scans = 1, conversion time = 2 ms.

3. High resolution mass spectra

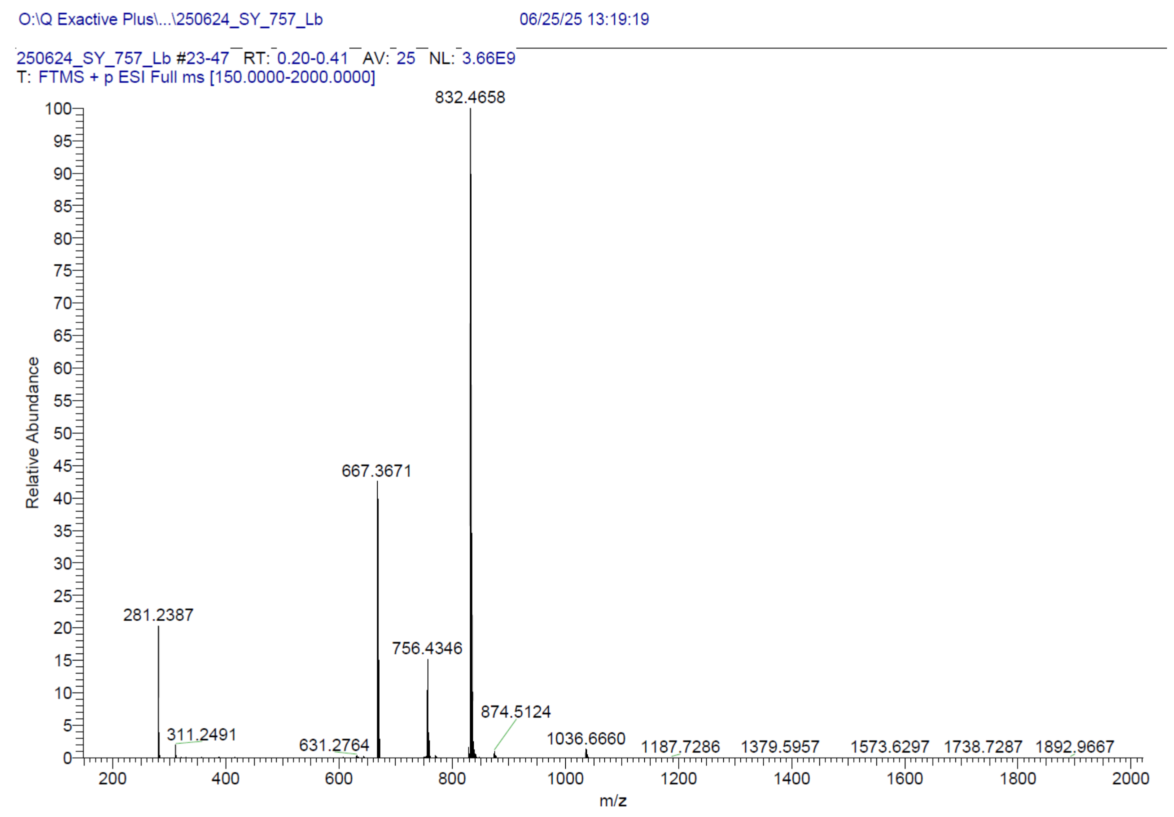

Figure S15. HRMS-ESI mass spectrum of [Pt(PCy$_3$)$_2$(Ph)(SbF$_6$)] (**4**).

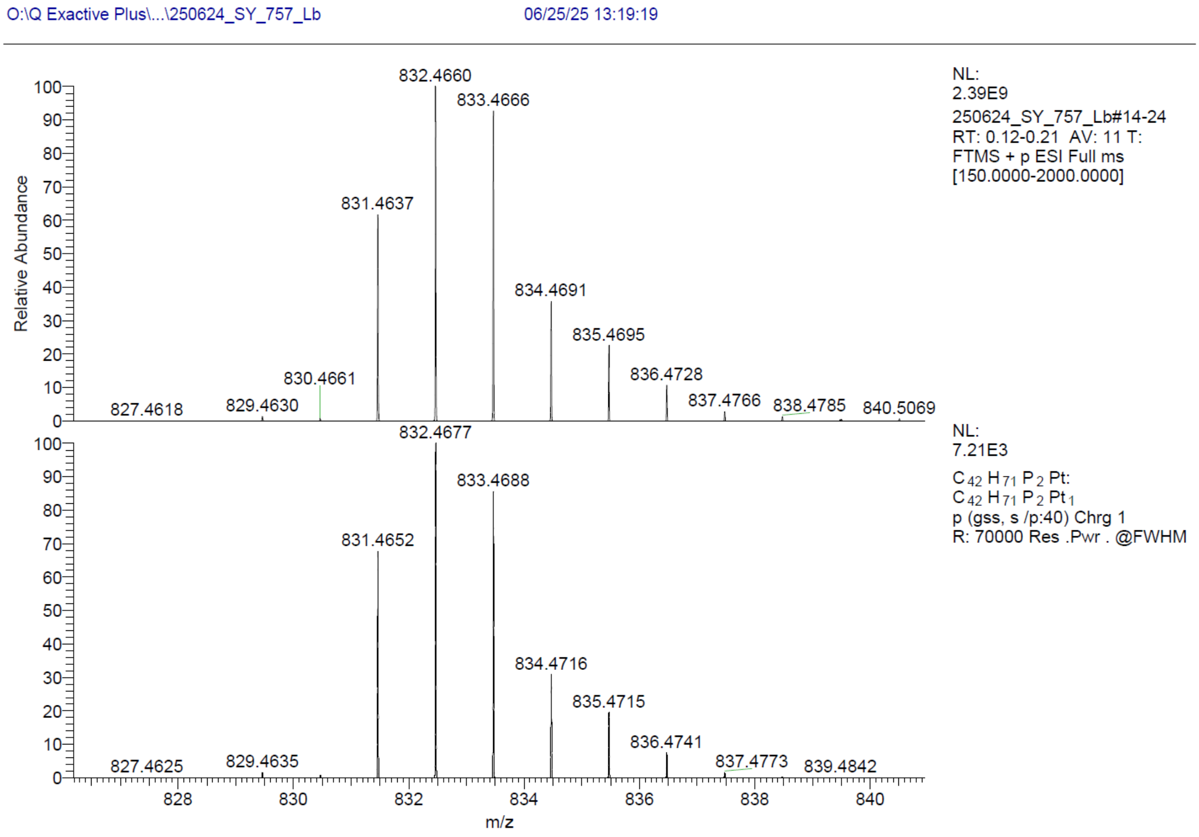

Figure S16. Top: HRMS-ESI mass spectrum of [Pt(PCy$_3$)$_2$(Ph)(SbF$_6$)] (**4**) enlarged at 832 m/z. Bottom: simulation of the signal at 832 m/z corresponding to [Pt(PCy$_3$)$_2$(Ph)]$^+$.

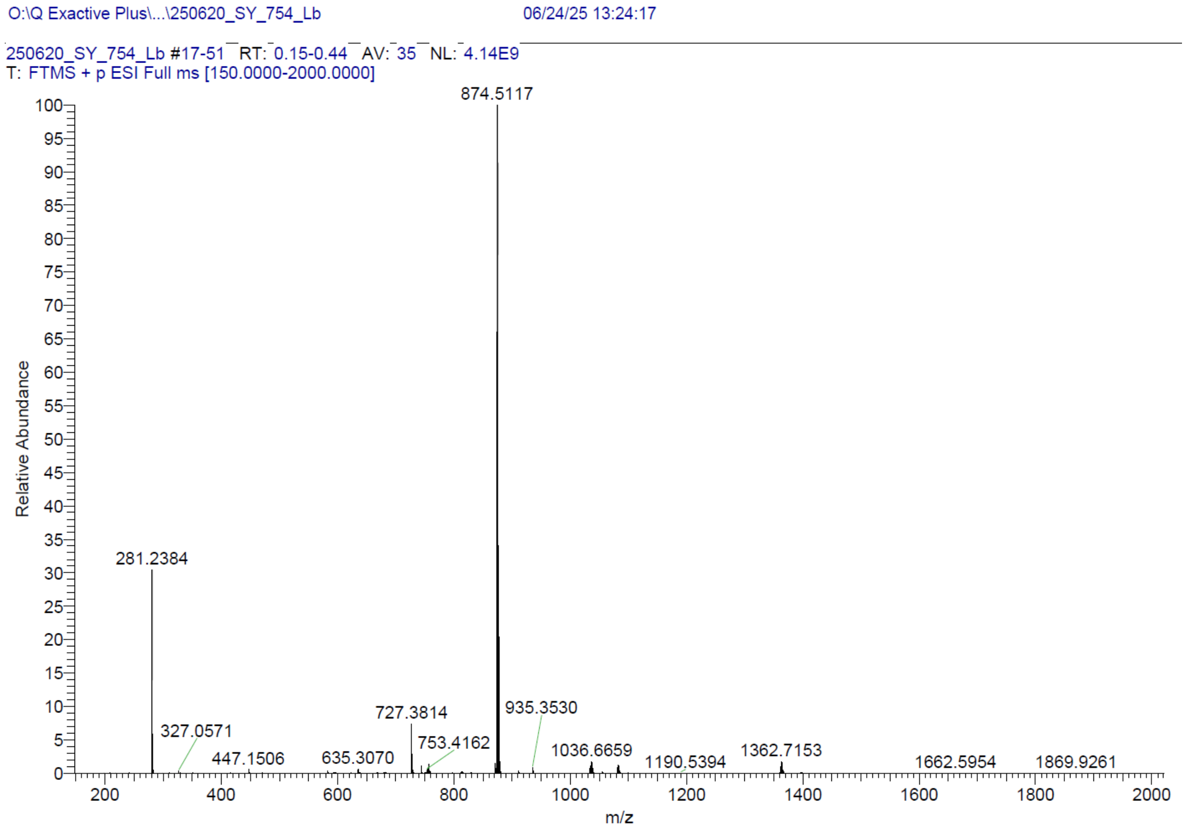

Figure S17. HRMS-ESI mass spectrum of [Pt(PCy$_3$)$_2$(Mes)(SbF$_6$)] (**5**).

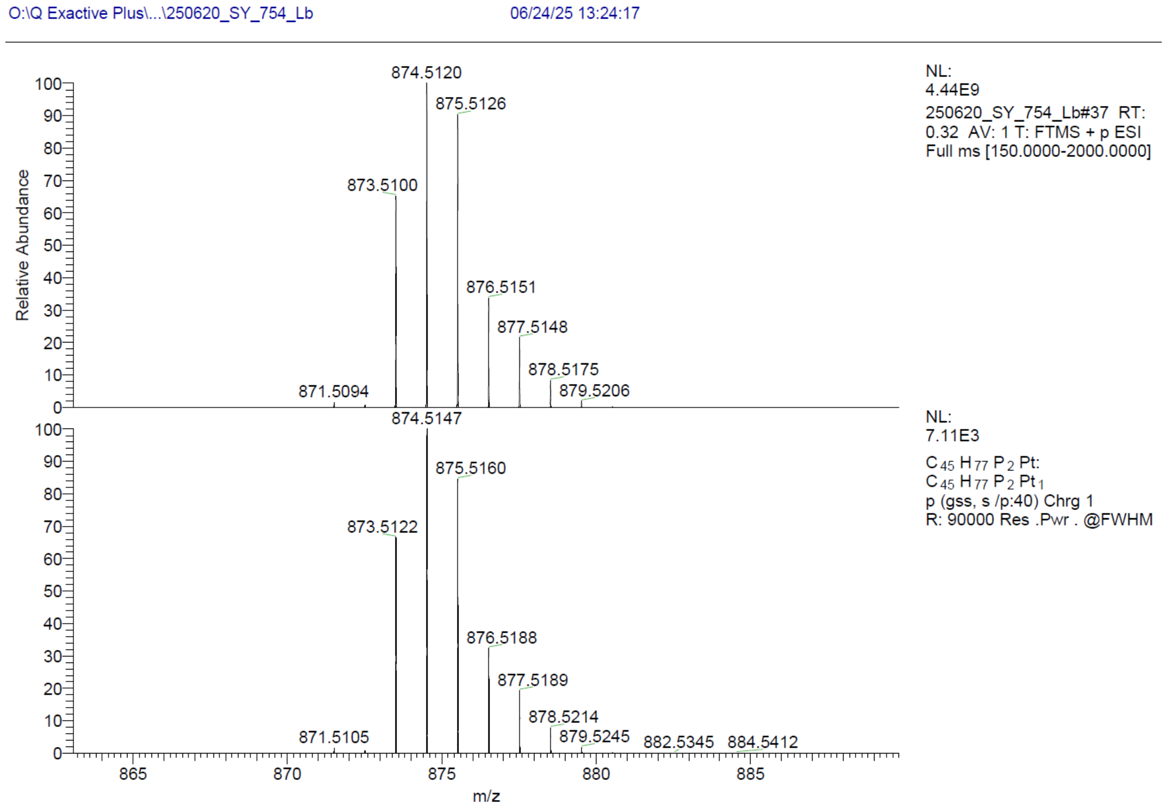

Figure S18. Top: HRMS-ESI mass spectrum of [Pt(PCy$_3$)$_2$(Mes)(SbF$_6$)] (**5**) enlarged at 874 m/z. Bottom: simulation of the signal at 874 m/z corresponding to [Pt(PCy$_3$)$_2$(Mes)]$^+$.

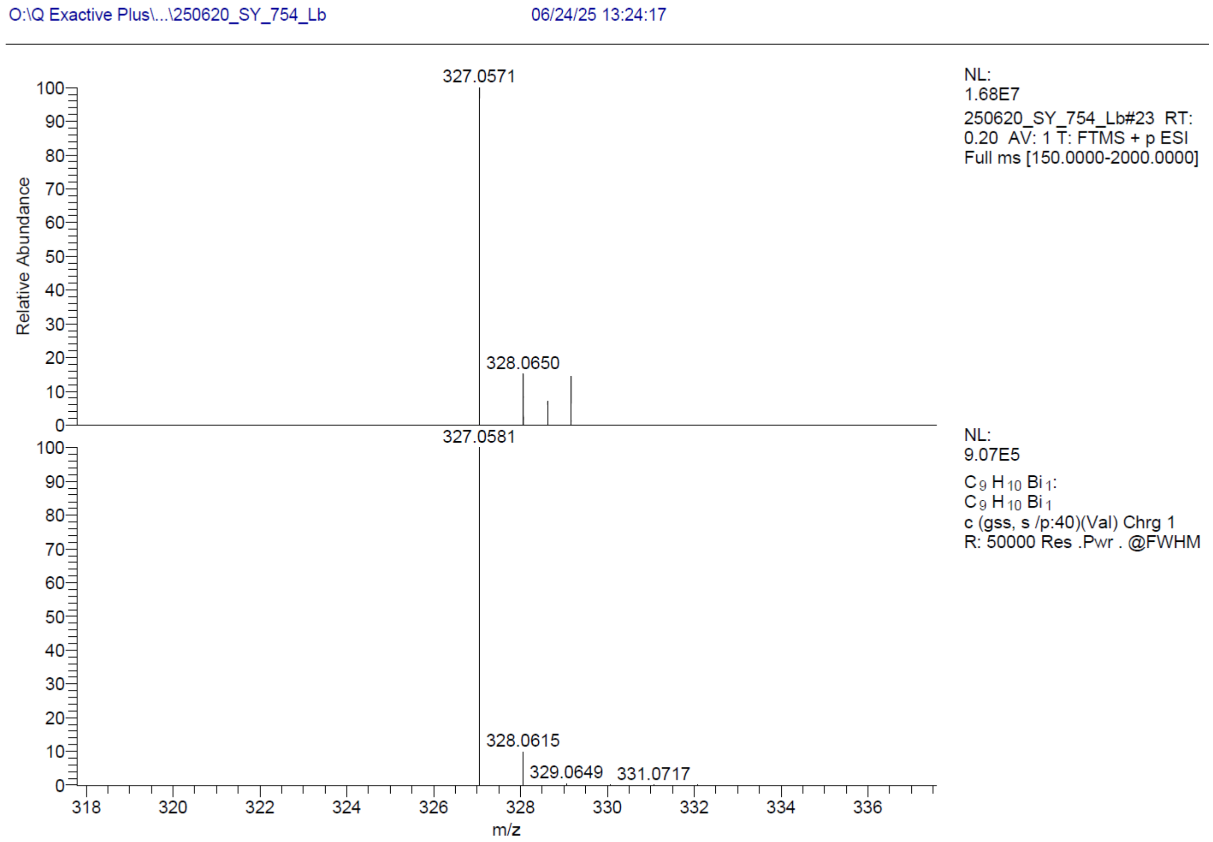

Figure S19: Top: HRMS-ESI mass spectrum obtained during monitoring of the reaction leading to $[Pt(PCy_3)_2(Mes)(SbF_6)]$ (**5**) enlarged at 327 m/z. Bottom: simulation of the signal at 327 m/z corresponding to $[BiMes–H^-]^+$.

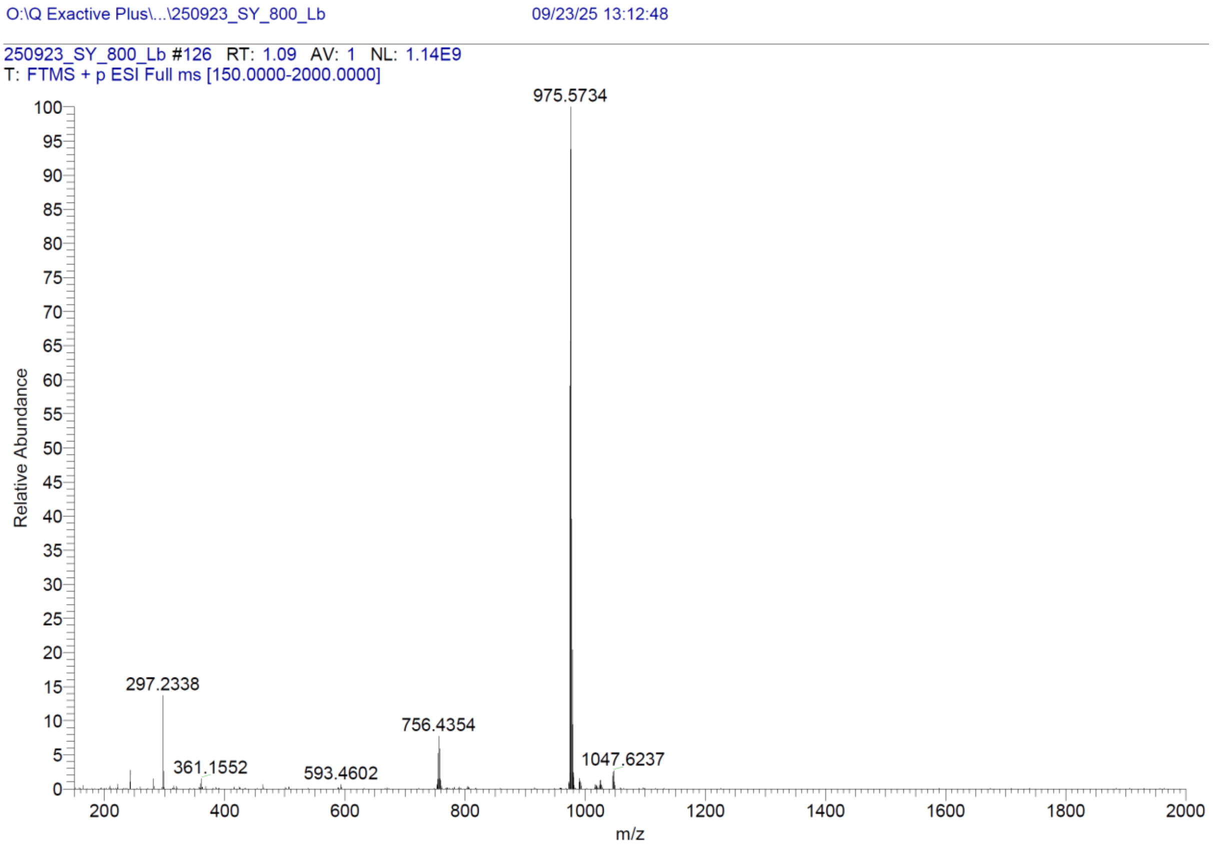

Figure S20. HRMS-ESI mass spectrum of $[Pt(PCy_3)_2(O_2\text{-}3,5\text{-}t\text{Bu}_2\text{-}C_6H_2)(SbF_6)]$ (**7**).

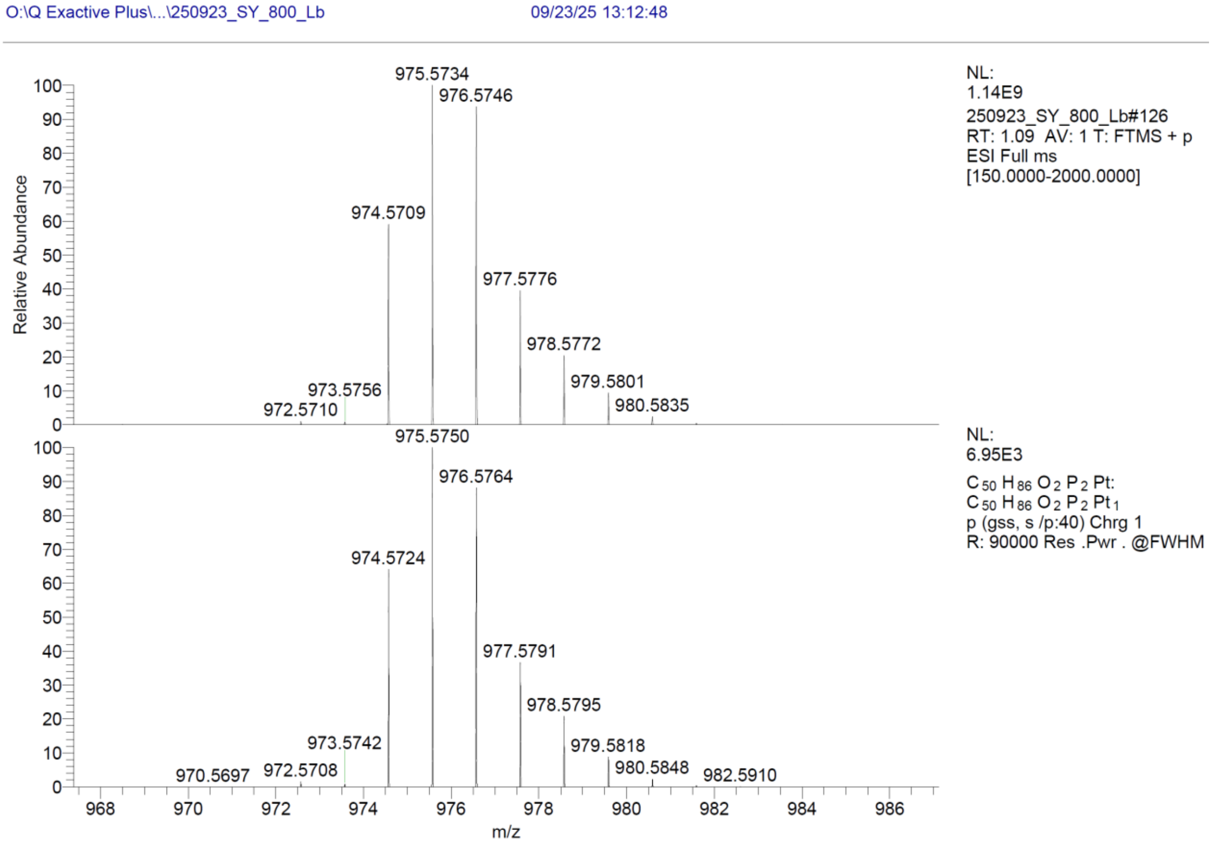

Figure S21. Top: HRMS-ESI mass spectrum of [Pt(PCy$_3$)$_2$(semiquinone)(SbF$_6$)] (**7**) enlarged at 975 m/z. Bottom: simulation of the signal at 975 m/z corresponding to [Pt(PCy$_3$)$_2$(O$_2$-3,5-*t*Bu$_2$-C$_6$H$_2$)]$^+$.

4. Cyclic voltammetry

BiMe₂SbF₆

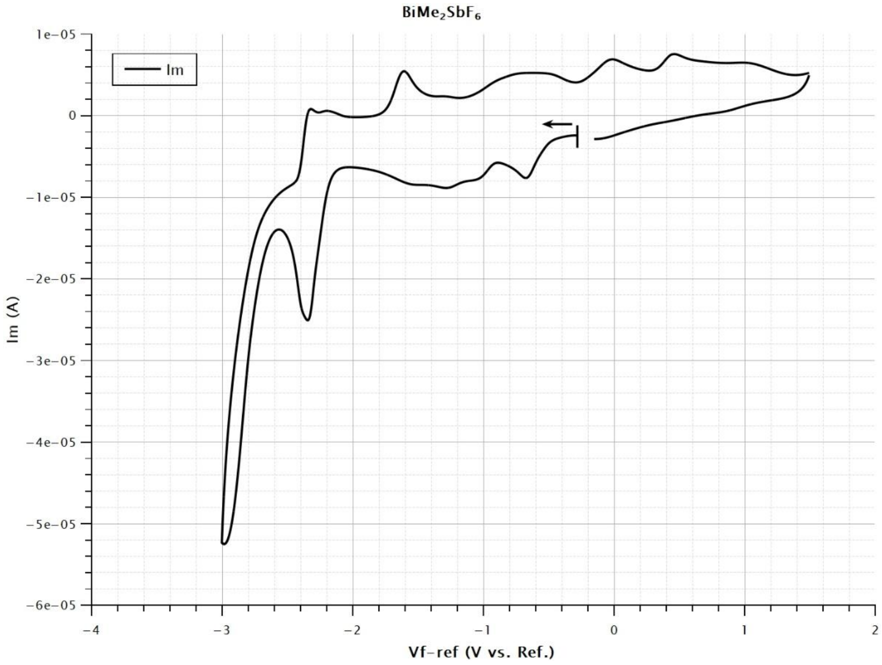

Figure S22. Cyclic voltammogram of [$BiMe_2(SbF_6)$] in MeCN (0.1 mol/L NBu_4PF_6) at a scan rate of 250 mV/s, referenced against ferrocene/ferrocenium couple.

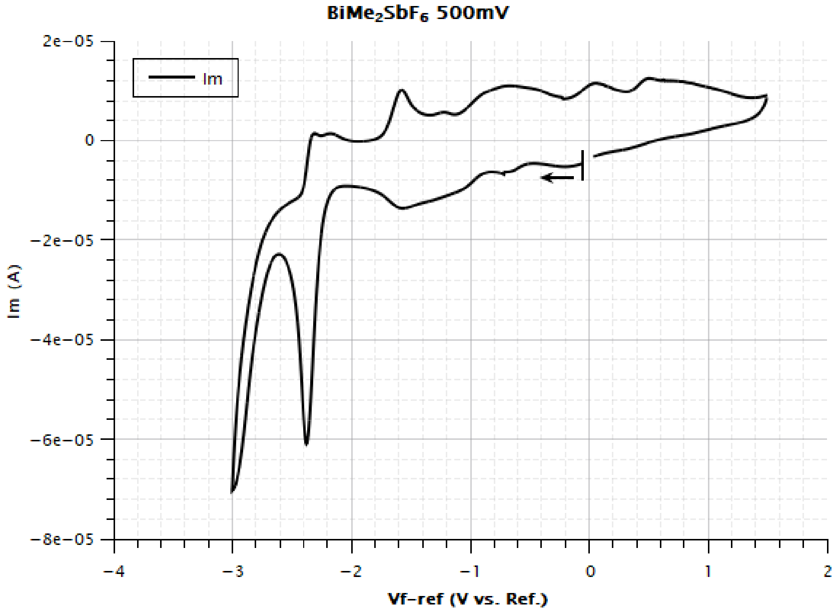

Figure S23: Cyclic voltammogram of [$BiMe_2(SbF_6)$] in MeCN (0.1 mol/L NBu_4PF_6) at a scan rate of 500 mV/s, referenced against ferrocene/ferrocenium couple.

$BiPh_2SbF_6$

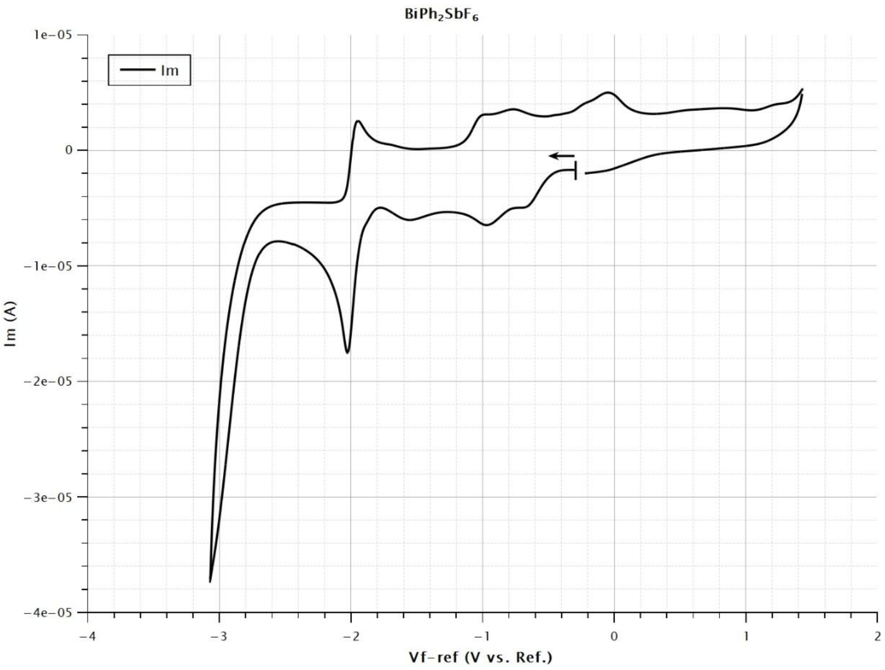

Figure S24. Cyclic voltammogram of [$BiPh_2(SbF_6)$] (**1**) in MeCN (0.1 mol/L NBu_4PF_6) at a scan rate of 250 mV/s, referenced against ferrocene/ferrocenium couple.

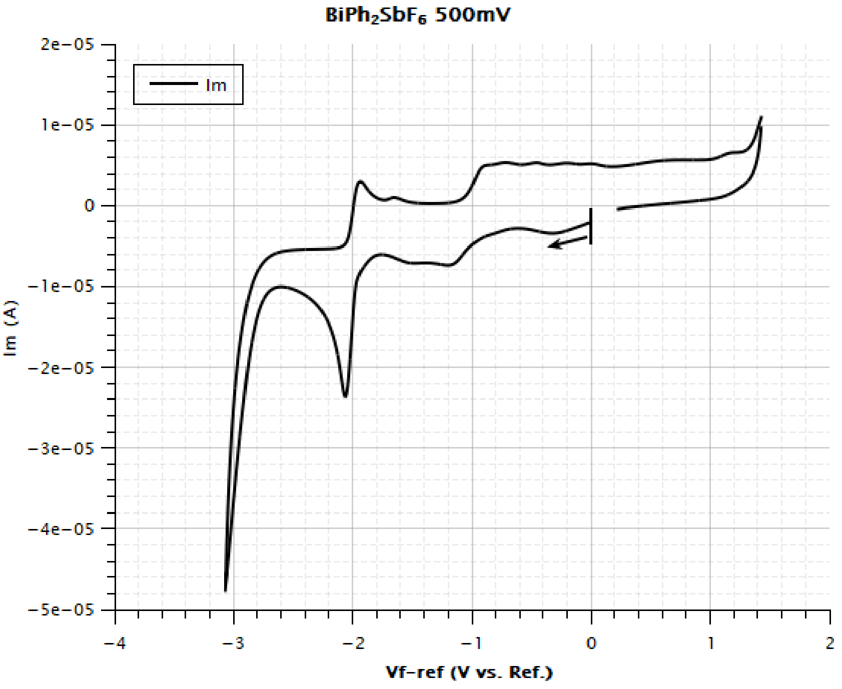

Figure S25: Cyclic voltammogram of [$BiPh_2(SbF_6)$] (**1**) in MeCN (0.1 mol/L NBu_4PF_6) at a scan rate of 500 mV/s, referenced against ferrocene/ferrocenium couple.

$BiMes_2SbF_6$

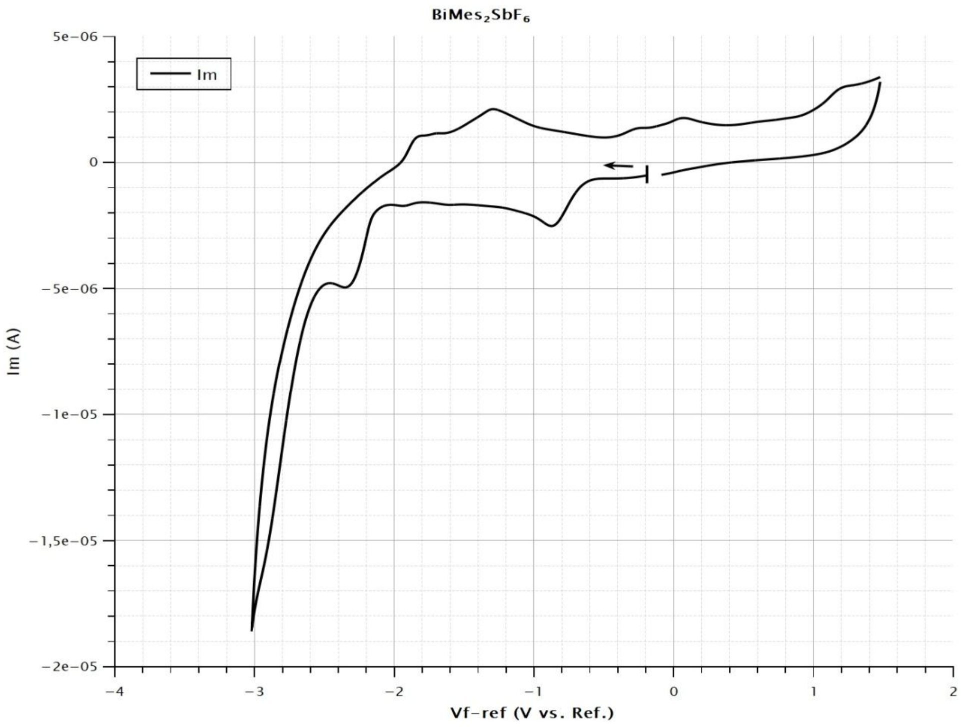

Figure S26: Cyclic voltammogram of $[BiMes_2(SbF_6)(thf)_2]$ (**2**) in MeCN (0.1 mol/L NBu_4PF_6) at a scan rate of 250 mV/s, referenced against ferrocene/ferrocenium couple.

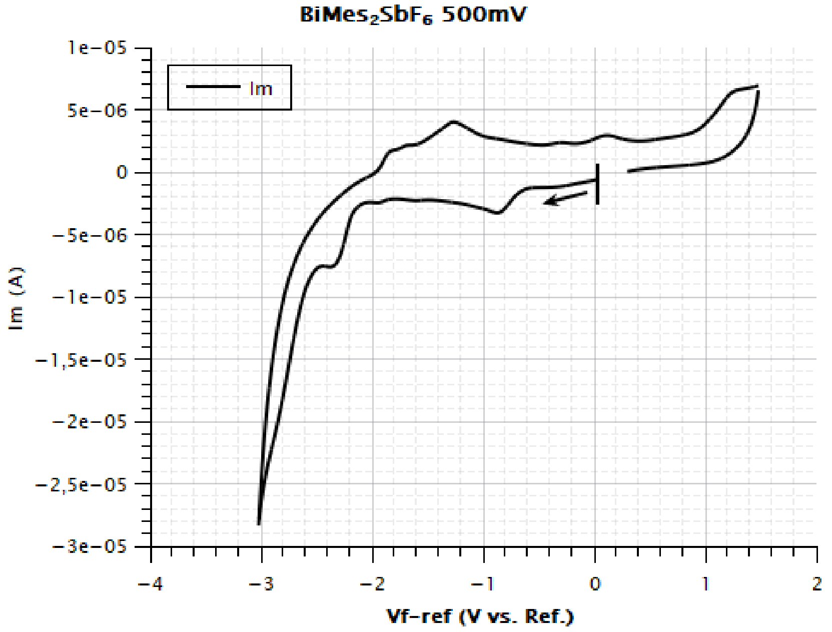

Figure S27: Cyclic voltammogram of $[BiMes_2(SbF_6)(thf)_2]$ (**2**) in MeCN (0.1 mol/L NBu_4PF_6) at a scan rate of 500 mV/s, referenced against ferrocene/ferrocenium couple.

$BiDipp_2SbF_6$

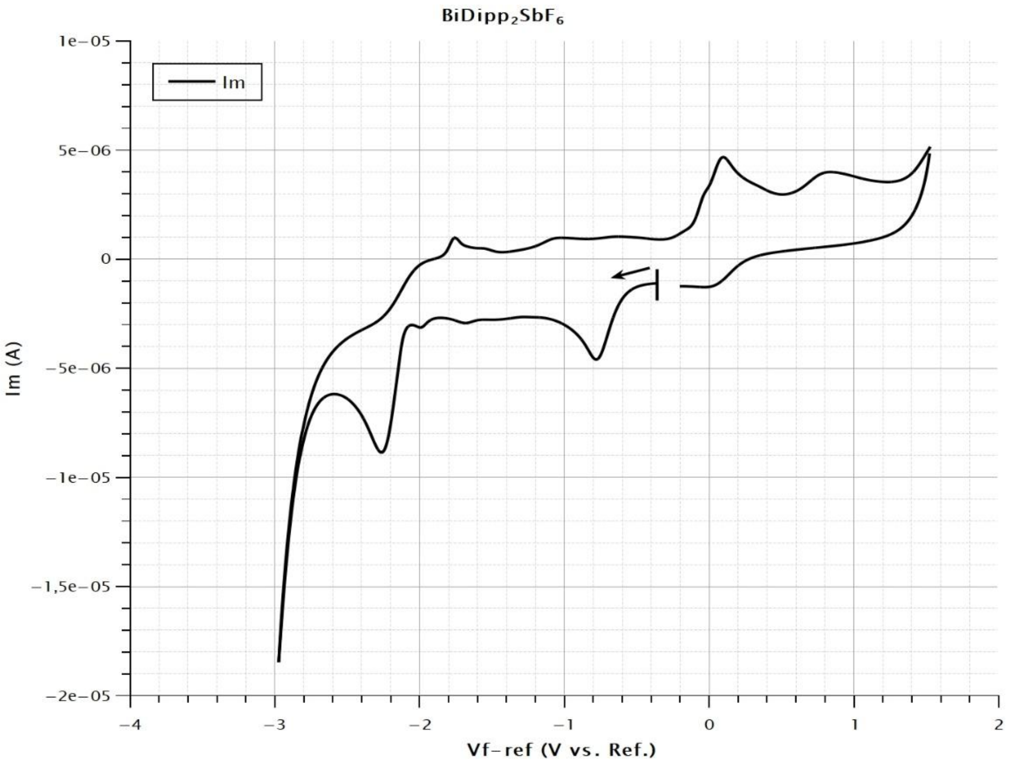

Figure S28: Cyclic voltammogram of $[BiDipp_2(SbF_6)(tol)]$ (**3**) in MeCN (0.1 mol/L NBu_4PF_6) at a scan rate of 250 mV/s, referenced against ferrocene/ferrocenium couple.

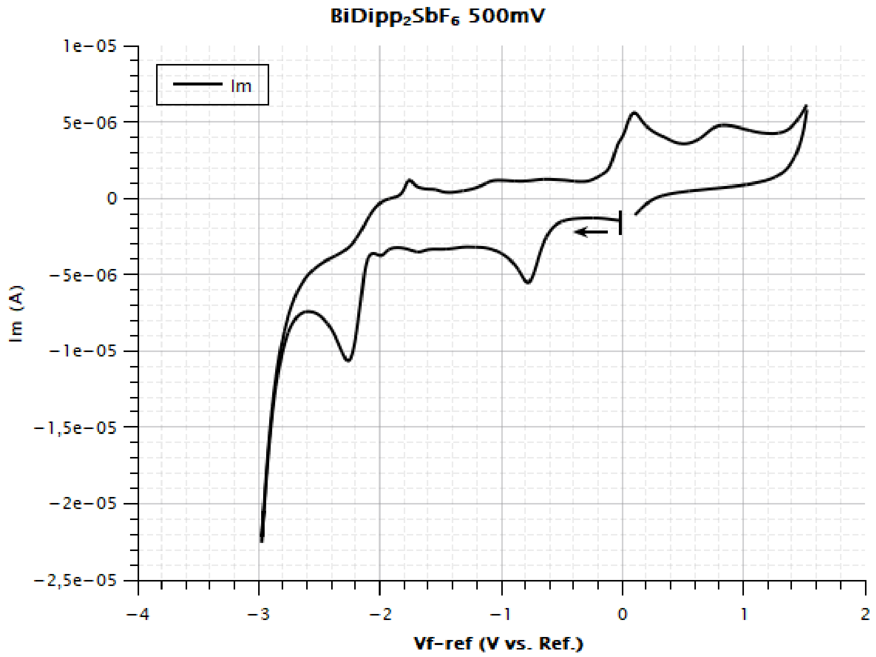

Figure S29: Cyclic voltammogram of $[BiDipp_2(SbF_6)(tol)]$ (**3**) in MeCN (0.1 mol/L NBu_4PF_6) at a scan rate of 500 mV/s, referenced against ferrocene/ferrocenium couple.

Cyclic voltammograms of compounds BiR_2SbF_6 (R = Me, Ph, Mes, Dipp) have been recorded in MeCN (0.1 mol/L NBu_4PF_6) at scan rates between 250 mV/s and 500 mV/s. When comparing these cyclic voltammograms, main features are observed at potentials between –2.34 ([$BiMe_2(SbF_6)$]) and –2.02 ([$BiPh_2(SbF_6)$] (**1**)) V vs Fc/Fc^+ and represent irreversible or partially reversible electrochemical events. The reversibility does not improve at higher scan rates, i.e. the irreversible chemical reactions take place at even higher rates. In the context of this work, the main conclusion that is to be drawn from the cyclic voltammetry data is the fact that compound [$BiMe_2(SbF_6)$] shows a more negative redox potential for the main electrochemical event than its aryl-substituted analogs.

5. UV-Vis spectra

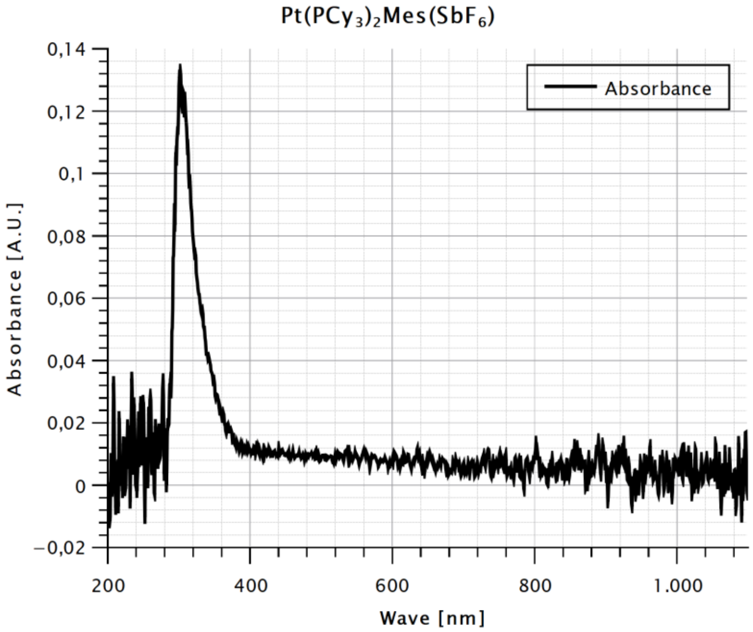

Figure S30: UV-Vis spectrum of [Pt(PCy_3)$_2$Mes(SbF_6)] (**5**) in 1,2-difluorobenzene (c = 7·10^{-4} mol/L). λ_{max} = 305 nm.

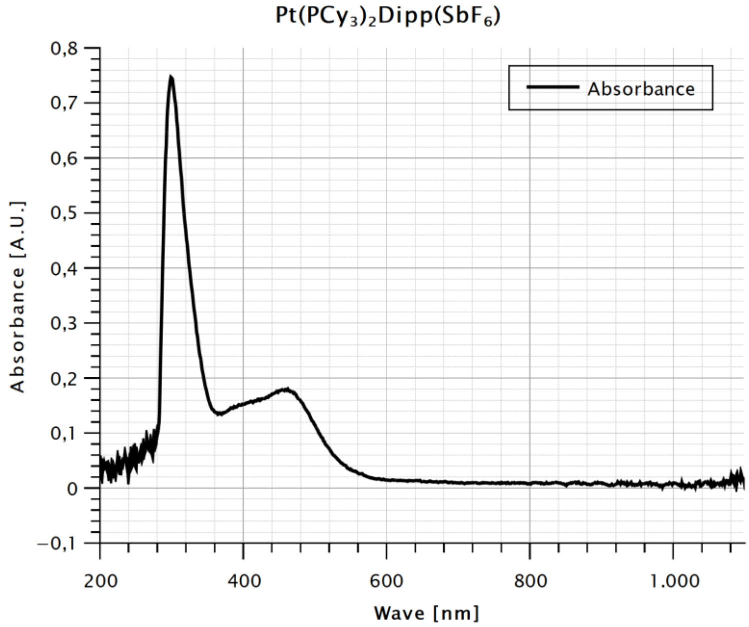

Figure S31: UV-Vis spectrum of [Pt(PCy_3)$_2$Dipp(SbF_6)] (**6**) in 1,2-difluorobenzene (c = 5·10^{-4} mol/L). λ_{max} = 299 nm, 462 nm.

6. Single-crystal X-ray diffraction analyses

Compound	**2**	**4**
Empirical formula	$C_{27}H_{40}BiCl_2F_6O_2Sb$	$C_{44}H_{75}Cl_4F_6P_2PtSb$
Formula weight	912.22	1238.62
Crystal color, habit	yellow, block	yellow, needle
Temperature/K	100	100
Crystal system	orthorhombic	triclinic
Space group	$Pca2_1$	$P\bar{1}$
a/Å	24.1052(7)	9.6049(5)
b/Å	15.4209(5)	12.2929(6)
c/Å	16.8727(6)	21.5269(10)
α/°	90	99.137(2)
β/°	90	92.117(2)
γ/°	90	101.358(2)
Volume/Å^3	6272.0(4)	2454.2(2)
Z	8	2
ρ_{calc}g/cm^3	1.932	1.676
μ/mm^{-1}	6.695	3.733
F(000)	3520.0	1240.0
Crystal size/mm	0.1x0.058x0.049	0.141x0.062x0.027
Diffractometer	Bruker D8 Quest	Bruker D8 Quest
Radiation	MoKα (λ = 0.71073)	MoKα (λ = 0.71073)
2Θ range for data collection/°	3.958 to 56.614	3.842 to 52.992
Reflections collected	104917	82622
Independent reflections	15563 [R_{int} = 0.0546]	10076 [R_{int} = 0.0597]
Data/restraint/parameters	15563/1/716	10076/0/523
Goodness-of-fit on F^2	1.029	1.049
Final R indexes [all data]	R_1 = 0.0357, wR_2 = 0.0444	R_1 = 0.0372, wR_2 = 0.0777
Final R indexes [I≥2σ(I)]	R_1 = 0.0281, wR_2 = 0.0429	R_1 = 0.0310, wR_2 = 0.0753
Largest diff. peak/hole/ e Å^{-3}	0.59/–0.80	1.38/–1.57
CCDC	2500345	2500346

Compound	**5**	**6**
Empirical formula	$C_{49.75}H_{84}F_6P_2PtSb$	$C_{48}H_{83}F_6P_2PtSb$
Formula weight	1174.94	1152.92
Crystal color, habit	orange, block	orange, plate
Temperature/K	100	100
Crystal system	Orthorhombic	monoclinic
Space group	$Cmc2_1$	$P2_1/c$
a/Å	14.3553(6)	12.8467(5)
b/Å	27.9613(11)	16.6475(6)
c/Å	13.4700(5)	22.9908(9)
α/°	90	90
β/°	90	91.9360(10)
γ/°	90	90
Volume/Å^3	5406.8(4)	4914.1(3)
Z	4	4
ρ_{calc}g/cm^3	1.443	1.558
μ/mm^{-1}	3.194	3.513
F(000)	2382.0	2336.0
Crystal size/mm	0.244x0.194x0.125	0.171x0.096x0.092
Diffractometer	Bruker D8 Quest	Bruker D8 Quest
Radiation	MoKα (λ = 0.71073)	MoKα (λ = 0.71073)
2Θ range for data collection/°	4.198 to 52.732	4.006 to 57.452
Reflections collected	54023	128779
Independent reflections	5615 [R_{int} = 0.0305]	12724 [R_{int} = 0.0325]
Data/restraint/parameters	5615/155/343	12724/0/527
Goodness-of-fit on F^2	1.124	1.035
Final R indexes [all data]	R_1 = 0.0175, wR_2 = 0.0452	R_1 = 0.0183, wR_2 = 0.0369
Final R indexes [I≥2σ(I)]	R_1 = 0.0168, wR_2 = 0.0449	R_1 = 0.0164, wR_2 = 0.0363
Largest diff. peak/hole/ e Å^{-3}	1.27/–0.49	1.12/–0.69
CCDC	2500347	2500348

Compound	**7**	**[BiMes$_2$(SbF$_6$)]**
Empirical formula	$C_{62}H_{94}F_{10}O_2P_2PtSb$	$C_{18}H_{22}BiF_6Sb$
Formula weight	1440.15	683.08
Crystal color, habit	colorless, needle	orange, plate
Temperature/K	100	100
Crystal system	monoclinic	monoclinic
Space group	$P2_1/n$	$P2_1/c$
a/Å	11.4872(5)	18.8530(18)
b/Å	16.9427(7)	7.3261(5)
c/Å	32.1044(12)	16.1353(17)
α/°	90	90
β/°	90.772(2)	115.338(7)
γ/°	90	90
Volume/Å^3	6247.7(4)	2014.2(3)
Z	4	4
ρ_{calc}g/cm^3	1.531	2.253
μ/mm^{-1}	2.791	10.118
F(000)	2924.0	1272.0
Crystal size/mm	0.109x0.104x0.055	0.12x0.08x0.05
Diffractometer	Bruker D8 Venture	STOE IPDS 2T
Radiation	MoKα (λ = 0.71073)	MoKα (λ = 0.71073)
2Θ range for data collection/°	4.284 to 52.044	4.782 to 51.732
Reflections collected	162771	28236
Independent reflections	12305 [R_{int} = 0.0513]	3877 [R_{int} = 0.0434]
Data/restraint/parameters	12305/78/734	3877/18/279
Goodness-of-fit on F^2	1.171	1.034
Final R indexes [all data]	R_1 = 0.0476, wR_2 = 0.0984	R_1 = 0.0469, wR_2 = 0.1257
Final R indexes [I≥2σ(I)]	R_1 = 0.0436, wR_2 = 0.0968	R_1 = 0.0399, wR_2 = 0.1212
Largest diff. peak/hole/ e Å^{-3}	1.69/–1.67	1.19/–1.56
CCDC	2500349	---

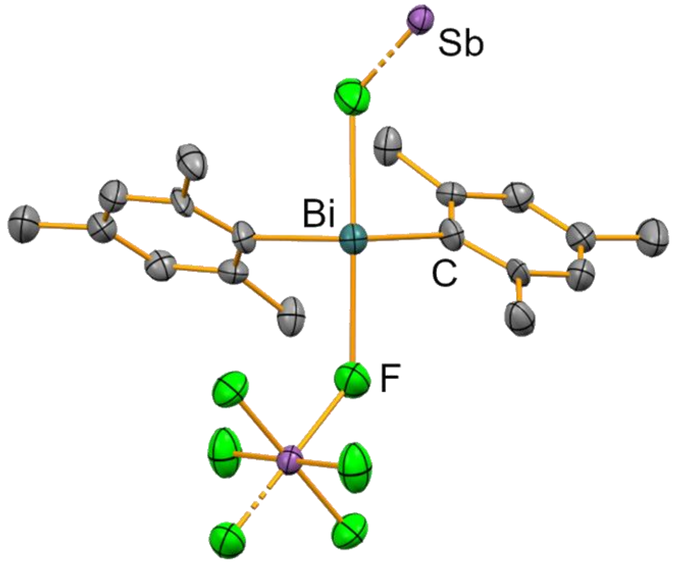

Figure S32: Molecular Structure of [$BiMes_2(SbF_6)$]. Displacement ellipsoids are drawn at 50% probability level. Hydrogen atoms are omitted for clarity. The picture shows one formula unit "[$BiMes_2(SbF_6)$]" plus one F and one Sb atom of an adjacent formula unit in order to showcase the formation of a one-dimensional coordination polymer in the solid state. The quality of the data set obtained from single-crystal X-ray diffraction experiments does not allow for a detailed discussion of bonding parameters, but establishes the connectivity of the molecular complex in the solid state.

7. Attempted trapping reactions (to trap suggested BiR intermediates)

The following reactions were conducted in 1,2-difluorobenzene (0.6 mL) using [Bi(Mes)$_2$(SbF$_6$)(thf)$_2$] (19 mg, 0.023 mmol, 1 eq.) or [Bi(Dipp)$_2$(SbF$_6$)(tol)] (20 mg, 0.023 mmol, 1 eq.) as bismuth cations, [Pt(PCy$_3$)$_2$] (17 mg, 0.023 mmol, 1 eq.) and 3,5-di-*tert*-butyl-1,2-benzoquinone (5 mg, 0.023 mmol, 1 eq.).

a) The quinone was added to the platinum complex first, resulting in a color change to bright yellow. After addition of the bismuth cation the color changed to dark green. The sample was subjected to ^{31}P NMR spectroscopic analysis and subsequent crystallization by layering the reaction mixture with *n*-pentane and storing at –30 °C.

b) The quinone was added to the bismuth cation, darkening the solution from yellow to red (for R = Mes) or from red to brown (for R = Dipp). Subsequent addition of [Pt(PCy$_3$)$_2$] leads to a color change to a brownish red in both cases. Each sample was subjected to ^{31}P NMR spectroscopic analysis and subsequent crystallization by layering the reaction mixture with *n*-pentane and storing at –30 °C.

c) The bismuth cation was added to the platinum precursor first and the quinone quickly thereafter, resulting in a similar brownish red color, as above. Each sample was subjected to ^{31}P NMR spectroscopic analysis and subsequent crystallization by layering the reaction mixture with *n*-pentane and storing at –30 °C.

The ^{31}P NMR spectra after the addition only show the formation of the [Pt(PCy$_3$)$_2$(R)(SbF$_6$)] complexes. Over time, the formation of a second signal at 47.2 ppm is observed, which is probably related to decomposition of the starting material and not related to the desired reaction. Crystallization led to the formation of [Pt(PCy$_3$)$_2$(O$_2$-3,5-*t*Bu$_2$-C$_6$H$_2$)(SbF$_6$)] in all cases. Thus, the attempted trapping of the suggested bismuthinidenes BiR (or oligomers thereof) was unsuccessful due to competing reaction pathways leading to compound **7**.

8. Computational details

All geometry optimizations and frequency analyses were performed using the Gaussian16[1] suite of programs. For compound **7**$^+$, the B3LYP+GD3/def2-TZVP level of theory was applied, while all other compounds were analyzed using geometry optimizations and frequency analyses on the B3LYP+GD3/def2-SVP level of theory followed by single point calculations on the B3LYP+GD3/def2-TZVP level of theory with a dichloromethane solvent model (PCM).[2–8] The cartesian coordinates of all geometry-optimized structures are provided in a separate file in xyz format (values in Å).

The computational analyses of 7$^+$ revealed energy values of $\Delta H = –2909.365487$ hartree and $\Delta G = –2909.517439$ hartree, which are reported here for completeness. The spin density plot of **7**$^+$ with two different orientations of the molecule is shown in Figure S33.

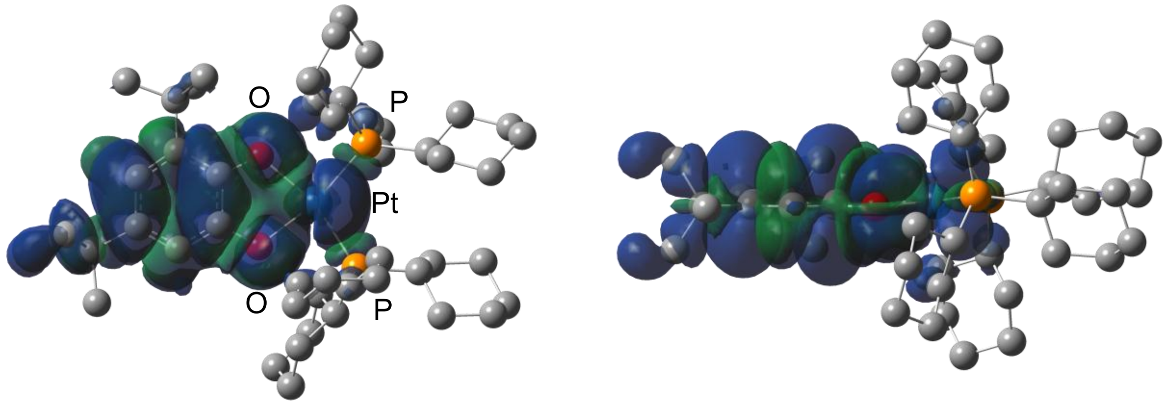

Figure S33: Top view (left) and side view (right) of the spin density distribution of $[Pt(PCy_3)_2(O_2\text{-}3,5\text{-}t\text{Bu}_2\text{-}C_6H_2)]^+$ (**7**$^+$) as determined by DFT calculations (isovalue = 0.0001; H atoms omitted for clarity).

Reactions of cationic bismuth species with Pt(PCy$_3$)$_2$ were analyzed computationally using the model compounds $[BiR_2]^+$ (R = Me, Ph) in order to gain additional insights into the differences between alkyl- and aryl-substituted species.

Previous experimental investigations have shown that the reaction of $Pt(PCy_3)_2$ with $[BiMe_2(SbF_6)]$ gives the adduct $[(PCy_3)_2Pt\rightarrow BiR_2]^+$,[9] while the experimental results described herein show that reactions of $Pt(PCy_3)_2$ with $[Bi(aryl)_2(L)_n(SbF_6)]$ give the products of oxidative aryl transfer $[(PCy_3)_2Pt(aryl)(SbF_6)]$ (L = neutral ligand, n = 0, 1, 2). In view of the more electron-donating character of the alkyl groups compared to the aryl groups, the lack of experimentally observable adducts between $Pt(PCy_3)_2$ and the complex cations $[Bi(aryl)_2]^+$ was surprising. In addition, Bi–C(aryl) bonds can be expected to be stronger than Bi–C(alkyl) bonds

according to the literature (mean Bi–C bond dissociation energy: 46.3 kcal/mol for $BiPh_3$ and 33.4 kcal/mol for $BiMe_3$).[10,11]

The energy values derived from computational analyses are summarized in Table S1. As a starting point it may be noted that for the selected cationic model compounds, $[BiMe_2]^+$ and $[BiPh_2]^+$, reactions with $Pt(PCy_3)_2$ to give products of oxidative group transfer, $[(PCy_3)_2PtR]^+$, along with 0.5 equivalents of RBi=BiR are thermodynamically viable for R = Me (ΔG = –30.9 kcal/mol) and R = Ph (ΔG = –39.4 kcal/mol). A more detailed analysis shows that adduct formation of $[BiR_2]^+$ with $Pt(PCy_3)_2$ is strongly exergonic (ΔG = –45.8 kcal/mol for $[BiMe_2]^+$; ΔG = –47.4 kcal/mol for $[BiPh_2]^+$).[12] This data is in agreement with the slightly more Lewis acidic character of the aryl species. We therefore suggest that adduct formation may indeed be the initiating step of the reaction between $Pt(PCy_3)_2$ and $[BiPh_2]^+$. The elimination of the bismuthinidene BiR (in its triplet state) from the adducts $[(PCy_3)_2Pt{\rightarrow}BiR_2]^+$ and subsequent transformations of the reactive intermediate BiR would be the next steps to be evaluated. In a conceptual approach we chose to analyze the formation of the experimentally observed Pt compounds $[(PCy_3)_2PtR]^+$ along with the bismuthinidenes BiR or the dibismuthenes RBi=BiR, which would still be highly reactive, but reasonable intermediates *en route* to the experimentally observed compounds BiR_3 (plus Bi^0). These reactions are endergonic due to the highly reactive nature of the suggested intermediates, but energetically more favorable in the case of the aryl species. For instance, the reaction of $[(PCy_3)_2Pt{\rightarrow}BiR_2]^+$ to give $[(PCy_3)_2PtR]^+$ and 0.5 equivalents of RBi=BiR shows Gibbs energies of ΔG = +14.8 kcal/mol for R = Me and ΔG = +8.1 kcal/mol for R = Ph. The subsequent redox disproportionation of PhBi=BiPh to yield $BiPh_3$ and Bi^0 would be the thermodynamic driving force of the overall reaction that is observed experimentally, which favors the observed reaction pathway over adduct formation. Kinetic parameters of the alkyl vs. aryl transfer from Bi to Pt have not been investigated in the context of this work.

Table S1. G_{corr} is the thermal correction to Gibbs free energy and $G_{298\ gas\ phase}$ is the sum of electronic and thermal free energies at the B3LYP+GD3/def2svp level of theory in gas phase (values in hartree); $E_{0\ DCM}$ is the electronic energy obtained at B3LYP+GD3/def2tzvp(PCM) level of theory in dichloromethane as the solvent (PCM) with the values given in hartree; $G_{298\ DCM}$ is the sum of $E_{0\ DCM}$ and G_{corr} (values in hartree).

Structure	G_{corr}	$G_{298\ gas\ phase}$	$E_{0\ DCM}$	$G_{298\ DCM}$
$Pt(PCy_3)_2$	0.892141	-2211.90247	-2214.55934	-2213.6672
$[BiMe_2]^+$	0.038867	-294.136651	-294.366412	-294.327545
[BiMe] (triplet)	0.00686	-254.553704	-254.615482	-254.608622
MeBi=BiMe	0.03537	-509.157844	-509.304924	-509.269554
$[(Cy_3P)_2Pt{\rightarrow}BiMe_2]^+$	0.957294	-2506.15662	-2509.02499	-2508.06769
$[(Cy_3P)_2PtMe]^+$	0.929364	-2251.55952	-2254.33862	-2253.40926
$[BiPh_2]^+$	0.138936	-677.274141	-677.987536	-677.8486
[BiPh] (triplet)	0.055366	-446.109225	-446.4243	-446.368934
PhBi=BiPh	0.131421	-892.271761	-892.923261	-892.79184
$[(Cy_3P)_2Pt{\rightarrow}BiPh_2]^+$	1.060536	-2889.27808	-2892.65187	-2891.59134
$[(Cy_3P)_2PtPh]^+$	0.978634	-2443.13159	-2446.16122	-2445.18258

9. References

(1) Gaussian 16, Revision A.03, Frisch, M. J.; Trucks, G. W.; Schlegel, H. B.; Scuseria, G. E.; Robb, M. A.; Cheeseman, J. R.; Scalmani, G.; Barone, V.; Petersson, G. A.; Nakatsuji, H.; Li, X.; Caricato, M.; Marenich, A. V.; Bloino, J.; Janesko, B. G.; Gomperts, R.; Mennucci, B.; Hratchian, H. P.; Ortiz, J. V.; Izmaylov, A. F.; Sonnenberg, J. L.; Williams-Young, D.; Ding, F.; Lipparini, F.; Egidi, F.; Goings, J.; Peng, B.; Petrone, A.; Henderson, T.; Ranasinghe, D.; Zakrzewski, V. G.; Gao, J.; Rega, N.; Zheng, G.; Liang, W.; Hada, M.; Ehara, M.; Toyota, K.; Fukuda, R.; Hasegawa, J.; Ishida, M.; Nakajima, T.; Honda, Y.; Kitao, O.; Nakai, H.; Vreven, T.; Throssell, K.; Montgomery, J. A., Jr.; Peralta, J. E.; Ogliaro, F.; Bearpark, M. J.; Heyd, J. J.; Brothers, E. N.; Kudin, K. N.; Staroverov, V. N.; Keith, T. A.; Kobayashi, R.; Normand, J.; Raghavachari, K.; Rendell, A. P.; Burant, J. C.; Iyengar, S. S.; Tomasi, J.; Cossi, M.; Millam, J. M.; Klene, M.; Adamo, C.; Cammi, R.; Ochterski, J. W.; Martin, R. L.; Morokuma, K.; Farkas, O.; Foresman, J. B.; Fox, D. J., Gaussian, Inc., Wallingford CT, 2016.

(2) Becke, A. D. Density-functional thermochemistry. III. The role of exact exchange. *J. Chem. Phys.* **1993**, *98*, 5648–5652.

(3) Lee, C.; Yang, W.; Parr, R. G. Development of the Colle-Salvetti correlation-energy formula into a functional of the electron density. *Phys. Rev. B, Condens. Matter* **1988**, *37*, 785–789.

(4) Vosko, S. H.; Wilk, L.; Nusair, M. Accurate spin-dependent electron liquid correlation energies for local spin density calculations: a critical analysis. *Can. J. Phys.* **1980**, *58*, 1200–1211.

(5) Stephens, P. J.; Devlin, F. J.; Chabalowski, C. F.; Frisch, M. J. Ab Initio Calculation of Vibrational Absorption and Circular Dichroism Spectra Using Density Functional Force Fields. *J. Phys. Chem.* **1994**, *98*, 11623–11627.

(6) Grimme, S.; Antony, J.; Ehrlich, S.; Krieg, H. A consistent and accurate ab initio parametrization of density functional dispersion correction (DFT-D) for the 94 elements H-Pu. *J. Chem. Phys.* **2010**, *132*, 154104.

(7) Weigend, F.; Ahlrichs, R. Balanced basis sets of split valence, triple zeta valence and quadruple zeta valence quality for H to Rn: Design and assessment of accuracy. *Phys. Chem. Chem. Phys.* **2005**, *7*, 3297–3305.

(8) Tomasi, J.; Mennucci, B.; Cammi, R. Quantum mechanical continuum solvation models. *Chem. Rev.* **2005**, *105*, 2999–3093.

(9) Schwarzmann, J.; Eskelinen, T.; Reith, S.; Ramler, J.; Karttunen, A. J.; Poater, J.; Lichtenberg, C. Bismuth as a Z-Type Ligand: an Unsupported Pt-Bi Donor-Acceptor Interaction and its Umpolung by Reaction with H_2. *Angew. Chem. Int. Ed.* **2024**, *63*, e202410291.

(10) Long, L. H.; Sackman, J. F. The heat of formation of bismuth trimethyl. *J. Chem. Soc., Faraday Trans.* **1954**, *50*, 1177.

(11) Steele, W. V. The standard enthalpies of formation of the triphenyl compounds of the Group V elements 2. Triphenylbismuth and the Ph–Bi mean bond-dissociation energy. *J. Chem. Thermodyn.* **1979**, *11*, 187–192.

(12) Note that qualitatively identical but numerically different results have been reported for the reaction of $Pt(PCy_3)_2$ with $[BiMe_2(SbF_6]$, using a different computational approach (ref. 9).
